# ABCB1 and ABCG2 Regulation at the Blood-Brain Barrier: Potential New Targets to Improve Brain Drug Delivery

**DOI:** 10.1124/pharmrev.120.000025

**Published:** 2023-09

**Authors:** Julia A. Schulz, Anika M.S. Hartz, Björn Bauer

**Affiliations:** Department of Pharmaceutical Sciences, College of Pharmacy (J.A.S., B.B.), Sanders-Brown Center on Aging and Department of Pharmacology and Nutritional Sciences, College of Medicine (A.M.S.H.), University of Kentucky, Lexington, Kentucky

## Abstract

The drug efflux transporters ABCB1 and ABCG2 at the blood-brain barrier limit the delivery of drugs into the brain. Strategies to overcome ABCB1/ABCG2 have been largely unsuccessful, which poses a tremendous clinical problem to successfully treat central nervous system (CNS) diseases. Understanding basic transporter biology, including intracellular regulation mechanisms that control these transporters, is critical to solving this clinical problem.

In this comprehensive review, we summarize current knowledge on signaling pathways that regulate ABCB1/ABCG2 at the blood-brain barrier. In Section I, we give a historical overview on blood-brain barrier research and introduce the role that ABCB1 and ABCG2 play in this context. In Section II, we summarize the most important strategies that have been tested to overcome the ABCB1/ABCG2 efflux system at the blood-brain barrier. In Section III, the main component of this review, we provide detailed information on the signaling pathways that have been identified to control ABCB1/ABCG2 at the blood-brain barrier and their potential clinical relevance. This is followed by Section IV, where we explain the clinical implications of ABCB1/ABCG2 regulation in the context of CNS disease. Lastly, in Section V, we conclude by highlighting examples of how transporter regulation could be targeted for therapeutic purposes in the clinic.

**Significance Statement:**

The ABCB1/ABCG2 drug efflux system at the blood-brain barrier poses a significant problem to successful drug delivery to the brain. The article reviews signaling pathways that regulate blood-brain barrier ABCB1/ABCG2 and could potentially be targeted for therapeutic purposes.

## I. Introduction

### A. The Blood-Brain Barrier

#### 1. History of Blood-Brain Barrier Knowledge

The discovery of the blood-brain barrier in 1885 by German microbiologist Paul Ehrlich was a serendipitous event that arose from Ehrlich’s work to determine the oxygen demand of the body (Ehrlich, 1885). In his experiments, Ehrlich injected rabbits intravenously with the “vital dyes” alizarin blue and indophenol blue and observed that all organs were stained by the dyes except for the brain. Several years later, in 1909, Ehrlich’s student Edwin Goldmann repeated the original studies in mice and rats to determine the organ distribution of the vital dye trypan blue (Goldmann, 1909). After intravenous injection, Goldmann observed that all peripheral organs were stained, albeit at a different staining intensity, while the cerebrospinal fluid (CSF) and all other parts of the central nervous system (CNS) remained unstained (Goldmann, 1909). Goldmann inferred such differences in dye distribution to be due to differences in secretion and architecture of the respective organs and concluded that a “physiological barrier membrane” (*Physiologische Grenzmembran*) separated the blood from the CNS. To test his hypothesis, Goldmann injected trypan blue into the mouse cranium and observed that the brain parenchyma and spinal cord were stained whereas the peripheral organs were not—the opposite effect of intravenous dye injection (Goldmann, 1913). This finding supported Goldmann’s hypothesis and provided further evidence for the existence of a barrier between the peripheral blood circulation and the CNS.

Today, Ehrlich’s and Goldmann’s experiments are considered the dawn of blood-brain barrier research. However, the term “blood-brain barrier” was not introduced until 1921 when Russian physiologist Lina Stern referred to it as *barrière hémato-encéphalique* (Stern and Gautier, 1921). Stern coined this term based on a series of experiments in which she injected guinea pigs, rabbits, cats, and dogs with a variety of substances, including bromides, strychnine, or bile salts, and then analyzed blood, CSF, and urine using colorimetric assays. After intravenous injection, Stern detected these substances only in blood and urine, whereas after intraventricular injection, she detected them only in the CSF. These results convinced Stern that the blood-brain barrier was not an anatomic but a functional structure that protects the CNS, prevents the uptake of toxic substances, and maintains normal physiologic conditions in the brain (Stern and Gautier, 1921).

In the years following these fundamental discoveries, a heated scientific controversy erupted over the nomenclature, location, and physiologic function of the blood-brain barrier. After much discussion, Hugo Spatz hypothesized in the early 1920s that barrier function must reside in the brain’s capillary endothelial cells (Spatz, 1934). Danish Nobel Laureate in Physiology or Medicine August Krogh argued that the blood-brain barrier could not be completely impermeable as Spatz had suggested years earlier, since Krogh’s own studies showed that nutrients and ions reached the brain parenchyma (Krogh, 1946). Definitive proof of blood-brain barrier location and function was provided by the seminal work of Reese and Karnovsky (1967) and Brightman and Reese (1969). These researchers intravenously injected mice, chicken, and goldfish with the enzyme horseradish peroxidase. Using electron microscopy, they showed in fixed brain slices that horseradish peroxidase remained confined in the lumen of brain microvessels due to brain endothelial tight junctions that prevented paracellular diffusion of the enzyme into the brain (Reese and Karnovsky, 1967; Brightman and Reese, 1969). These findings unequivocally demonstrated that the tight junctions that had been identified between brain capillary endothelial cells several years earlier by Muir and Peters (1962) restrict paracellular diffusion of solutes across the blood-brain barrier (Reese and Karnovsky, 1967; Brightman and Reese, 1969).

#### 2. The Neurovascular Unit

**Fig. 1:**
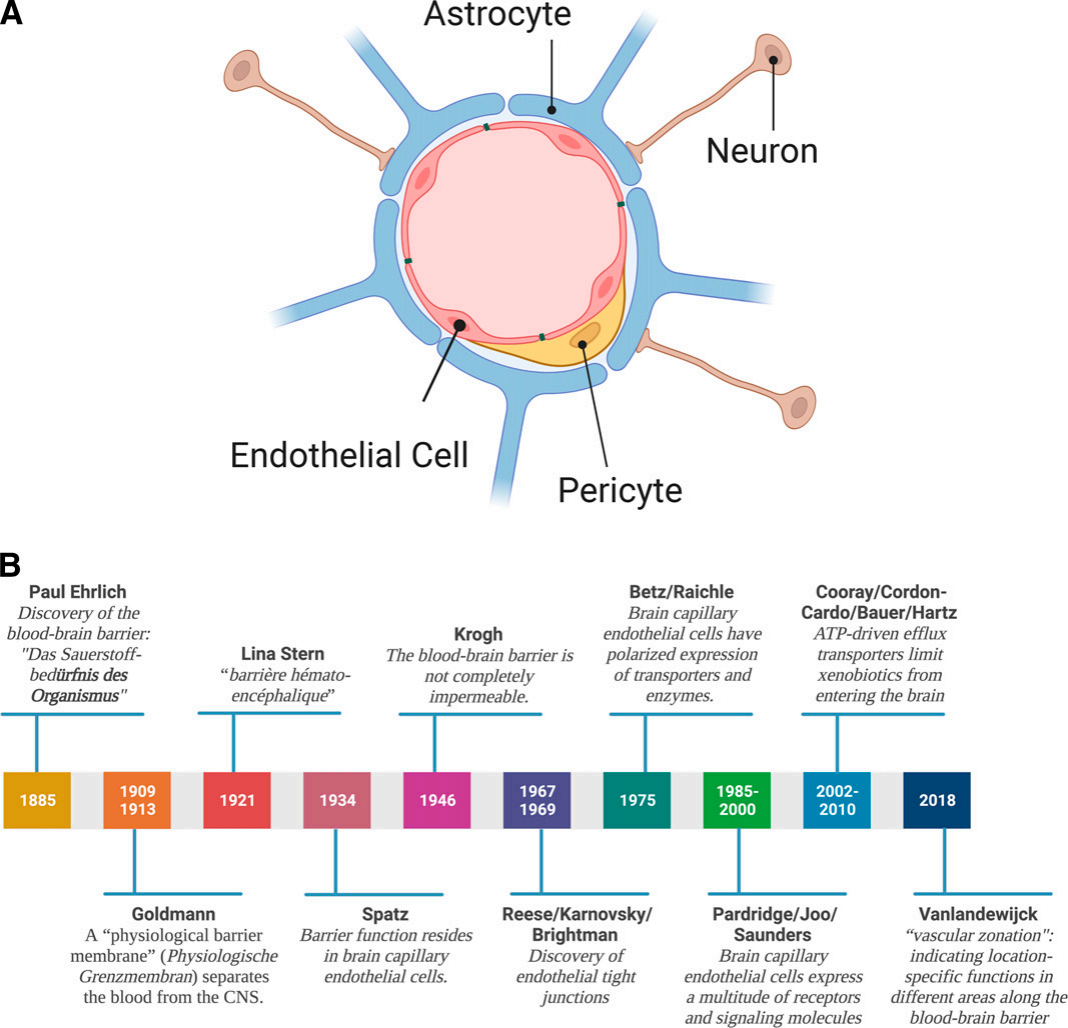
(A) The neurovascular unit. The neurovascular unit consists of endothelial cells surrounded by a basement membrane, astrocytes, pericytes, and neurons. This four-cell structure also known as the “neurovascular unit” is responsible for the regulation of blood-brain barrier function. (B) History of the blood-brain barrier. Timeline of fundamental discoveries made in the blood-brain barrier field. Created with BioRender.com.

In the early 2000s, a new concept arose: Barrier function is not solely based on endothelial cell properties but rather relies on the anatomic and functional interaction of endothelial cells with pericytes, astrocytes, and neurons. Together, these cells form an anatomically complex and functionally highly regulated and dynamic multicell structure referred to as the “neurovascular unit” (Fig. 1; Stroke Progress Review Group, 2002).

In the neurovascular unit, brain capillary endothelial cells form the first layer of barrier function. Characteristics of brain capillary endothelial cells that contribute to barrier function include high expression levels of tight junction proteins, lack of fenestration, low pinocytic activity, and a large number of mitochondria that provide ATP to support a high energy demand (Reese and Karnovsky, 1967; Brightman and Reese, 1969; Oldendorf and Brown, 1975; Oldendorf et al., 1977; Betz and Goldstein, 1978; Betz et al., 1980; Yoshida et al., 1988). Brain capillary endothelial cells also have polarized expression of transporters and enzymes. Alkaline phosphatase and other enzymes localize to the luminal membrane facing the blood, while Na^+^/K^+^-ATPase and the Na^+^-dependent small amino acid carrier are located in the abluminal membrane, facing the brain. This polar protein expression is the prerequisite for directional transport across the blood-brain barrier and has been described for glucose and several amino acids, including leucine and isoleucine (Betz et al., 1975; Raichle et al., 1975). Moreover, protein expression also changes along the vascular continuum of the blood-brain barrier, a phenomenon recently described as “vascular zonation,” indicating location-specific functions in different areas along the vasculature of the blood-brain barrier (Vanlandewijck et al., 2018). Brain capillary endothelial cells are surrounded by a basement membrane that consists of collagens and laminins, as well as proteins involved in extracellular matrix and basement membrane reorganization, e.g., matrix metalloproteinases (Bonnans et al., 2014; Joutel et al., 2016). The basement membrane provides structure and support for endothelial cells and is involved in signal transduction between brain capillary endothelial cells and brain parenchymal cells (Hynes, 2009; Baeten and Akassoglou, 2011; Nehra et al., 2022). Embedded within the basement membrane are pericytes (multifunctional mural cells), the second cell type of the neurovascular unit, that cover the abluminal brain capillary surface (Cuevas et al., 1984; Armulik et al., 2005, 2010; Winkler et al., 2011; Sweeney et al., 2016). Depending on the brain region, pericytes cover 20% to 99% of the abluminal surface of brain capillaries (Mathiisen et al., 2010; Hartmann et al., 2015; Herndon et al., 2017; Berthiaume et al., 2018). Astrocytes, the third cell type of the neurovascular unit, have end feet that sit on top of the basement membrane and cover approximately 60% of the abluminal surface area of brain capillaries (Wolff, 1963; Mathiisen et al., 2010; Korogod et al., 2015). The fourth and last cell type at the neurovascular unit is neurons. Neurons interact with the neurovascular unit either through astrocytic connections or through direct interaction of interneurons with endothelial cells (Niwa et al., 2000; Gotoh et al., 2001).

Together, the neurovascular unit, consisting of endothelial cells, pericytes, astrocytes, and neurons, maintains brain homeostasis, protects the CNS from neurotoxic compounds, and is responsible for communication between the periphery and the CNS.

##### a. Communication at the neurovascular unit

The neurovascular unit represents a critical blood-brain interface that ensures regulated bidirectional communication between the periphery and the CNS (Stern and Gautier, 1921; Terrando et al., 2011; Marchi et al., 2013; Chen et al., 2020) and is a highly regulated anatomic structure that senses and responds to information flowing from the periphery to the brain and vice versa (Wyss-Coray and Rogers, 2012; Chen et al., 2020).

To enable communication, cells of the neurovascular unit are highly specialized and equipped with a myriad of signaling molecules. For example, brain capillary endothelial cells express a multitude of receptors and signaling molecules (Stefanovich, 1979; Karnushina et al., 1980; Joo, 1985, 1993; Pardridge et al., 1985). Among these proteins are signaling molecules like cAMP (Stefanovich, 1979; Karnushina et al., 1980), insulin receptor (Frank and Pardridge, 1981; Pardridge et al., 1985), hormone receptors (Edvinsson and Owman, 1975), as well as receptor tyrosine kinases like platelet-derived growth factor receptor (Smits et al., 1989). Additionally, enzymes involved in the synthesis and degradation of signaling molecules like cyclooxygenase (Baba et al., 1985) and phosphodiesterases (Stefanovich, 1979) are also present at the blood-brain barrier (Joo, 1985; 1993; Saunders et al., 2014). These signaling molecules enable endothelial cells to communicate between the periphery and the brain.

Pericytes are in direct, basolateral contact with brain capillary endothelial cells, which allows for direct communication between the two cell types through gap junctions (Cuevas et al., 1984; Armulik et al., 2005, 2010; Winkler et al., 2011; Sweeney et al., 2016). Pericytes play a vital role in blood-brain barrier development and maintenance of barrier function. In this regard, loss of pericyte function results in abnormal capillary development and increased capillary permeability (Hellström et al., 2001; Armulik et al., 2010).

Astrocytes cover a large area of the basolateral side of brain capillary endothelial cells and, therefore, are in an ideal position to communicate with brain capillary endothelial cells and regulate barrier function. Signaling from astrocytes to brain capillary endothelial cells is essential for development of tight junctions and localization of transporters and other endothelial proteins. One such regulatory pathway involves the signaling peptide sonic hedgehog that is released by astrocytes. After secretion, sonic hedgehog binds to the Patch-1 receptor on brain capillary endothelial cells inducing downstream activation of the transcription factor GLI family zinc finger 1 (Alvarez et al., 2011, 2013). Activation of GLI family zinc finger 1, in turn, increases the expression of the tight junction proteins claudin 5 and occludin, decreasing blood-brain barrier permeability (Alvarez et al., 2011). Astrocytes are uniquely located between endothelial cells and neurons and enable communication between those two cell types (Niwa et al., 2000; Gotoh et al., 2001). Astrocyte-endothelial cell communication is referred to as neurovascular coupling, indicating the close interaction of neuronal activity and cerebral blood flow. Cerebral blood flow is selectively increased in areas with high neuronal activity to compensate for higher energy consumption (Cox et al., 1993; Chaigneau et al., 2003). Additionally, interneurons regulate cerebral blood flow by releasing vasoactive molecules, such as prostaglandins or nitric oxide (NO) (Niwa et al., 2000; Gotoh et al., 2001; Iadecola, 2017). Together, cells of the neurovascular unit work together to ensure effective communication among themselves as well as the periphery and the brain.

#### 3. Barrier Function

Barrier function is pivotal for protecting and ensuring nutrient supply to the brain. The blood-brain barrier achieves this through tightly regulated interplay among enzymes, transporters, and structural proteins that cooperate through four different mechanisms. First, tight junction proteins form a physical barrier by sealing off paracellular pathways, which prevents passive diffusion of hydrophilic endo- and xenobiotics (Reese and Karnovsky, 1967; Brightman and Reese, 1969; Saunders et al., 2014). Second, metabolic enzymes expressed in endothelial cells form a metabolic barrier by degrading, and thereby deactivating, CNS-active drugs before they can reach their targets (Ghersi-Egea et al., 1994; Dauchy et al., 2009; Saunders et al., 2017). Third, influx transporters facilitate the uptake of specific nutrients like glucose and amino acids (Oldendorf, 1971; Daneman et al., 2010; Saunders et al., 2017). These transporters belong to the solute carrier superfamily and are either facilitative, secondary, or tertiary active transporters (Deng et al., 2014; Morris et al., 2017; Yan et al., 2019b). Fourth, ATP-driven efflux transporters export metabolic waste and limit xenobiotics, including a myriad of therapeutic drugs, from entering the brain (Cordon-Cardo et al., 1989; Cooray et al., 2002; Hartz et al., 2005, 2009, 2010b; Bauer et al., 2006; Hartz and Bauer, 2010; Miller, 2010; Saunders et al., 2017). Notably, influx and efflux transporters make up approximately 15% of blood-brain barrier–specific proteins indicating a high relevance for proper barrier function (Li et al., 2001; Shusta et al., 2002; Pardridge, 2007; Kamiie et al., 2008; Uchida et al., 2011; Uchida et al., 2014).

### B. ATP-Binding Cassette Efflux Transporters

Efflux transporters belong to the ATP-binding cassette (ABC) transporter superfamily of primary active transporters and are organized based on their gene structure, amino acid sequence, and phylogenetic analyses into seven subfamilies (ABCA–ABCG; Sarkadi et al., 2006; Robey et al., 2018). ABC transporter structure and function are conserved across the different subfamilies as well as across multiple species, including fungi, bacteria, protozoa, insects, fish, and mammals (Klokouzas et al., 2003; Kovalchuk and Driessen, 2010; Gebhard, 2012; Luckenbach et al., 2014; Kowalski et al., 2020). The human genome contains 48 different ABC transporters (Vasiliou et al., 2009; Morris et al., 2017; Robey et al., 2018); 19 of these transporters are expressed in the CNS, most of them at the blood-brain barrier (Hartz and Bauer, 2011). Common structural features of ABC transporters include two transmembrane domains (TMD) and two nucleotide-binding domains (NBD); (Loo et al., 2002). The TMDs form the substrate binding pocket and facilitate substrate movement across the blood-brain barrier and other membranes, while the NBDs hydrolyze ATP to provide the energy for active substrate movement against a concentration gradient (Loo et al., 2002). The NBD structure is highly conserved across the seven ABC subfamilies and across species. Common motifs, including the Walker A (G-x(4)-GK-[TS]) and B ([RK]-x(3)-G-x(3)-LhhhD) motifs, are preserved throughout all ABC transporters (Hyde et al., 1990; Dean et al., 2001). The TMD sequences on the other hand are highly variable, which allows a broad, diverse substrate spectrum that includes lipophilic drugs, hydrophilic metabolites, glucuronides, and sulfate conjugates (Dean et al., 2001; de Vries et al., 2007; Kruh et al., 2007). The two most prominent efflux transporters at the blood-brain barrier, P-glycoprotein (P-glycoprotein nucleotide-binding domains; ABCB1) and breast cancer resistance protein (BCRP; ABCG2), are the main topic of this review and are discussed in detail later.

#### 1. History of ABCB1

**Fig. 2:**
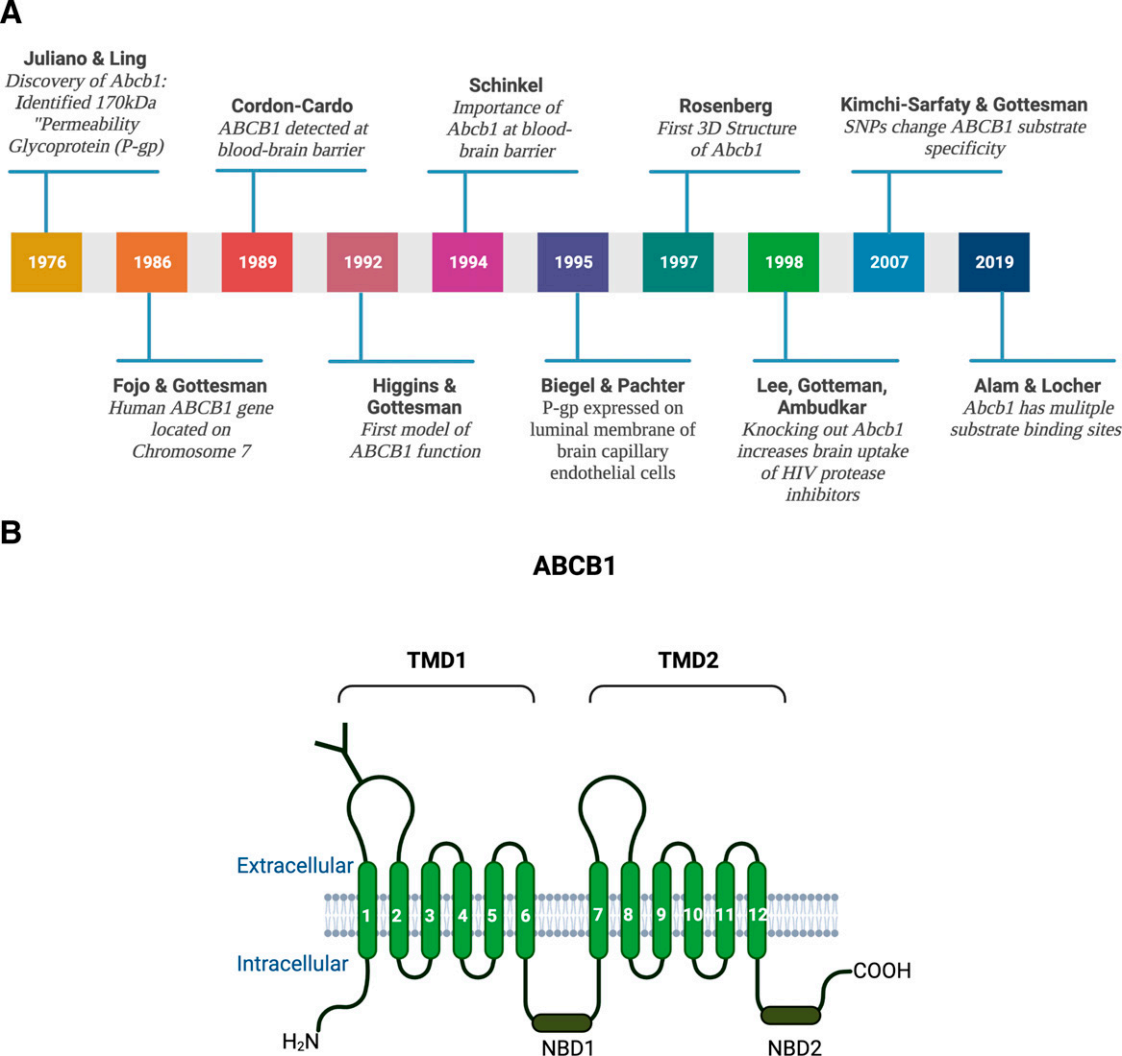
(A) History of ABCB1. From the discovery of the “permeability glycoprotein” by Juliano and Ling in 1976 to structural insights into substrate and inhibitor discrimination by human ABCB1 revealed by Alam and Locher in 2019. (B) ABCB1 structure. ABCB1 consists of two transmembrane domains TMD1 and TMD2, each of which has six transmembrane spanning *α*-helices and a nucleotide binding domain (NBD1 and NBD2). ABCB1 is N-glycosylated at the first extracellular loop. Created with BioRender.com.

The development of mustard gas derivatives, antimetabolites, and antibiotics as anticancer drugs in the 1940s and 1950s significantly improved the survival of cancer patients (Goodman et al., 1946; Hitchings and Elion, 1954; Pinkel, 1959; DeVita and Chu, 2008). Success in treatment, however, also revealed that patients could be resistant to anticancer drugs (Law, 1952; Niero et al., 2014). Work analyzing this drug resistance in bacterial and mammalian cells eventually led to the discovery of ABCB1 and other ABC transporters (Fig. 2).

In 1970, June Biedler postulated that resistance to actinomycin D and other anticancer drugs in Chinese hamster ovary cells was caused by changes in cell permeability (Biedler and Riehm, 1970). Biedler’s experiments showed that multidrug resistance was established through stable chromosome changes, possibly in membrane-related genes (Biedler and Riehm, 1970). Later, Juliano and Ling determined that the expression of a 170 kDa glycoprotein in the membrane of drug-resistant Chinese hamster ovary cells correlated with the level of drug resistance (Juliano and Ling, 1976). Since the protein was not expressed in wild-type cells and drug resistance corresponded with changes in drug permeation, Juliano and Ling postulated that this new glycoprotein changed the permeability of the cell membrane and, therefore, named it “permeability glycoprotein” or P-glycoprotein [old nomenclature: PGY1, MDR1, CLCS; current: ABCB1 (human protein), Abcb1a/Abcb1b (rodent proteins), *Abcb1a/Abcb1b* (rodent genes), *ABCB1* (human gene) (Juliano and Ling, 1976)]. Homolog genes and proteins were later detected in bacteria as well as in mice and humans, where the gene is located on chromosome 7 (chromosome 5 in mice; Chen et al., 1986; Fojo et al., 1986; Gros et al., 1986; Callen et al., 1987).

In human samples, ABCB1 localizes to the apical surface of epithelial and endothelial cells of excretory and barrier organs and tissues such as liver, kidney, intestine, colon, and placenta, suggesting a role in secretion, elimination, and protection (Thiebaut et al., 1987; Croop et al., 1989). The role of ABCB1 in protecting critical organs was further demonstrated when it was detected at the human blood-brain barrier in 1989 (Cordon-Cardo et al., 1989). At the blood-brain barrier, ABCB1 is expressed in the luminal membrane of brain capillary endothelial cells (Biegel et al., 1995). Even though ABCB1 had been identified at the blood-brain barrier, its role and significance were initially obscure. Based on tissue distribution and expression in drug-resistant cancer cells, the leading hypothesis was that ABCB1 was involved in the active excretion of toxic xenobiotics and metabolites from the brain and other excretory tissues. To test the physiologic role of Abcb1, Schinkel et al. developed an *Abcb1a* (originally referred to as *mdr1a*) knockout mouse (Schinkel et al., 1994). Shortly after establishing this unique knockout mouse, unexpected circumstances led to a serendipitous finding. Due to a mite infestation of Schinkel’s mouse colonies, all animals—wild-type and *Abcb1a* knockout—were treated with ivermectin, a standard veterinary anthelmintic drug. After treatment, all *Abcb1a* knockout mice, presented with paralytic symptoms and died from neurotoxicity; however, none of the wild-type mice died. Toxicological testing showed that *Abcb1a* knockout mice had 90-fold higher ivermectin brain levels compared with wild-type or heterozygous littermates, which correlated with a 100-fold increase in sensitivity to ivermectin-induced neurotoxicity (Schinkel et al., 1994). Today, Abcb1 deficiency is well recognized in dogs and cats and is routinely screened for in pets to prevent ivermectin-induced toxicity (Roulet et al., 2003; Mealey et al., 2023). Based on their observations in mice, Schinkel and coworkers concluded that blood-brain barrier Abcb1a was important for protecting the brain and creating a pharmacological sanctuary. This was further corroborated by results from Kim et al. showing that knocking out *Abcb1a* increased oral absorption and brain uptake of human immunodeficiency virus (HIV) protease inhibitors (Kim et al., 1998a,b; Lee et al., 1998). Combined, these findings indicated that Abcb1a acts as a double-edged sword at the blood-brain barrier: on the one hand, Abcb1a-mediated efflux is vital for protecting the brain; on the other hand, Abcb1a prevents uptake of potentially CNS-active drugs, significantly limiting their CNS efficacy.

At that time in the 1990s, initial models were proposed to explain ABCB1-mediated transport function. The original model hypothesized a central pore that facilitates active substrate expulsion through the apical plasma membrane (Borst and Schinkel, 1997). However, the first 3D structure for ABCB1 proposed by Rosenberg and colleagues showed that ABCB1 is closed toward the cytoplasm side, contradicting the pore model (Rosenberg et al., 1997). A second model attempting to explain ABCB1 transport function, referred to as the “hydrophobic vacuum cleaner model,” postulates that a substrate moves laterally through the membrane until ABCB1 removes it through a flipping process, indicating that ABCB1 acts as a flippase (Higgins and Gottesman, 1992; Gottesman and Pastan, 1993). Additional data from experiments to elucidate ABCB1 structure showed a central, polyspecific substrate binding chamber that is accessible from the cytoplasm as well as the lipid membrane, suggesting that ABCB1 efflux function is most likely based on a combination of both models (Rosenberg et al., 2005; Aller et al., 2009). However, understanding ABCB1 function is further complicated by several synonymous single nucleotide polymorphisms (SNPs) that are inconsequential for ABCB1 protein structure but affect function and substrate binding (Kimchi-Sarfaty et al., 2007; Fung and Gottesman, 2009; Dickens et al., 2013; Hattori et al., 2018). Recently, Alam et al. found multiple substrate binding pockets in the ABCB1 molecule and concluded that the pocket a compound binds to determines if this compound is an ABCB1 substrate or inhibitor (Alam et al., 2019). In addition, Dastvan et al. (2019) demonstrated that ABCB1 substrate binding decreases the activation energy for ATP hydrolysis and showed that ATP hydrolysis must occur before or simultaneously to substrate translocation (Dastvan et al., 2019). To this date, exactly how ABCB1 functions remains unclear, and more research is necessary to fully elucidate the mechanism of ABCB1-mediated efflux transport.

#### 2. History of ABCG2

**Fig. 3:**
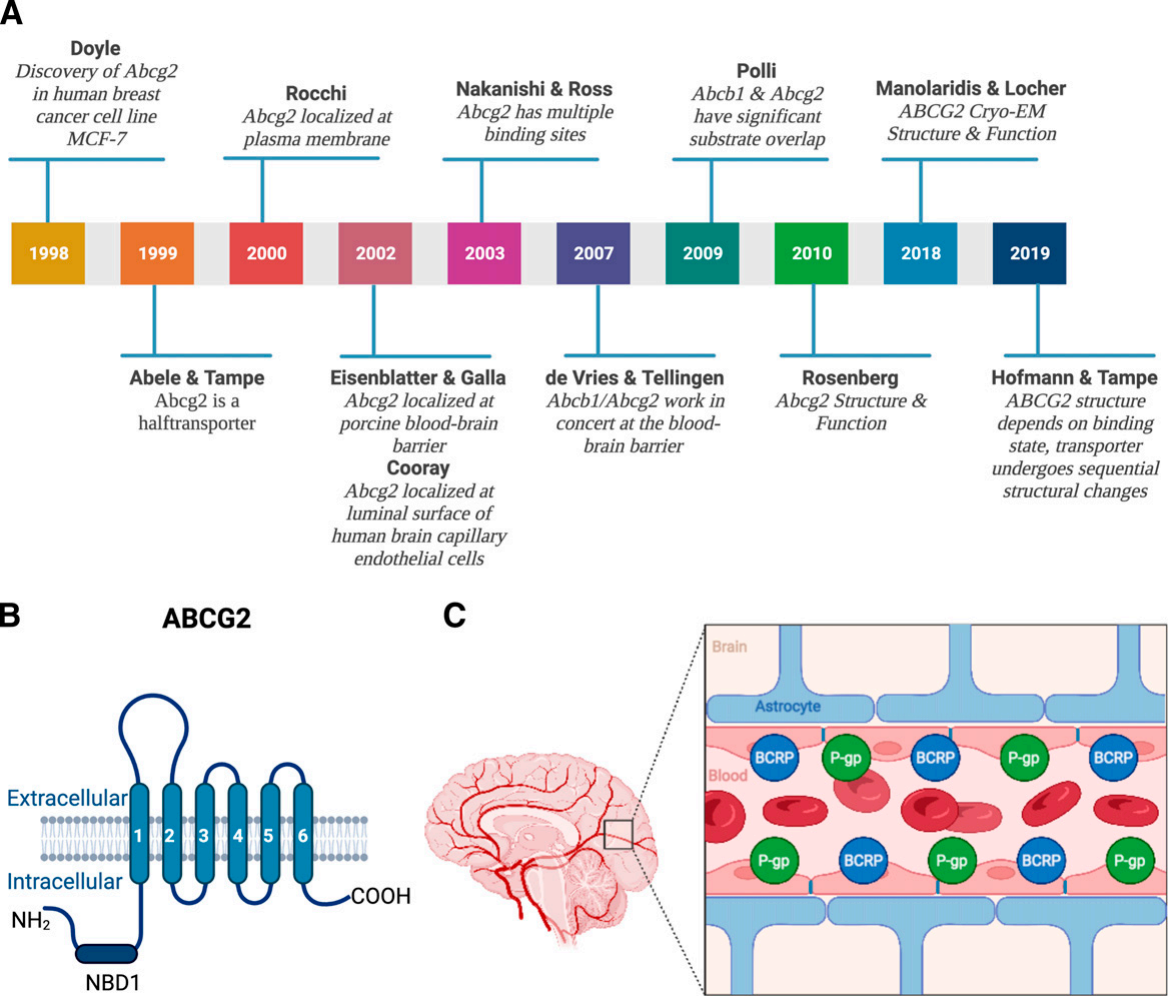
(A) History of ABCG2. From the discovery of the “breast cancer resistance protein” ABCG2 in 1998 to its cryo-EM structure and function. (B) ABCG2 structure. ABCG2 consists of one transmembrane domain that has six transmembrane spanning *α*-helices and one nucleotide binding domain (NBD1). ABCG2 is a half transporter that needs to homodimerize to fully function. (C) ABCB1 and ABCG2 at the blood-brain barrier. ABCB1 and ABCG2 are both located at the luminal membrane of endothelial cells comprising the blood-brain barrier. They act as a “first line of defense” by limiting xenobiotics including a large number of therapeutic drugs from entering into the brain. Created with BioRender.com.

In 1992, Nakagawa et al. discovered that exposing MCF-7 human breast cancer cells to the ABCB1 inhibitor verapamil did not reverse mitoxantrone resistance (Nakagawa et al., 1992). Moreover, daunorubicin and rhodamine 123 efflux from MCF-7 cells was not affected by the ABCB1 inhibitor cyclosporin A but was reversed by depleting ATP (Lee et al., 1997). These data indicated the existence of another active, ATP-driven efflux transporter in cancer cells. In 1998, Doyle et al. (Doyle et al., 1998) compared gene expression in parental versus doxorubicin-resistant MCF-7 cells and revealed a differentially expressed messenger RNA (mRNA) that coded for a new ABC transporter: breast cancer resistance protein [old nomenclature: EST157481, MXR, BCRP, ABCP, CD338; current: ABCG2 (human protein), Abcg2 (rodent protein), *Abcg2* (rodent gene), *ABCG2* (human gene; Fig. 3A; Doyle et al., 1998)].

Physiologically, ABCG2 is expressed in barrier organs and tissues including the blood-brain barrier, where ABCG2 localizes to the luminal plasma membrane of endothelial cells and facilitates directional efflux across the blood-brain barrier from brain to blood (Cooray et al., 2002; Eisenblätter and Galla, 2002; Zhang et al., 2003). However, in contrast to other ABC transporters, *ABCG2* codes for only one transmembrane domain with one nucleotide binding site, resulting in a protein of approximately 70 kDa, which is half the size of other ABC transporters. Therefore, ABCG2 is a so-called half transporter that needs to homodimerize to fully function (Fig. 3B; Abele and Tampe, 1999; Rocchi et al., 2000; Kage et al., 2002).

Structural studies, homology modeling, and transport studies with ABCG2 mutants identified multiple substrate binding sites that confirmed an overlapping substrate spectrum with ABCB1 (Nakanishi et al., 2003; Clark et al., 2006; Xu et al., 2007; Rosenberg et al., 2010). The exact mechanism of transport function, however, was unknown until Manolaridis et al. (2018) recently constructed cryo-electron microscopy structures of ABCG2 in substrate- and ATP-bound pre- and post-translocation states. These different conformations revealed that substrates bind to a central, hydrophobic binding pocket that faces the cytoplasm. Upon ATP binding and hydrolysis, a conformational shift collapses the substrate binding pocket, which opens an external cavity and pushes the substrate across the membrane and out of the cell (Manolaridis et al., 2018). Hofmann et al. (2019) confirmed these findings and found two distinct cryo-electron microscopy structures of an ABCG2 bacterial homolog. Based on these structures, the authors determined that transporter conformation depends on substrate- and ATP-binding state and suggested sequential conformation changes during the transport process. In addition to exogenous drug transport, ABCG2 has also been implicated in the transport of endogenous metabolites including estrogens, steroids, and folates (Imai et al., 2003; Suzuki et al., 2003; Ifergan et al., 2004, 2005).

Like *ABCB1*, several SNPs in *ABCG2* have been identified in patients (Zamber et al., 2003; Furukawa et al., 2009; Delord et al., 2013). For example, Allegra et al. (2018) recently demonstrated that the SNP 1194 + 928 rs13120400 T>C (position 89033527), an intronic variant of *ABCG2*, is associated with decreased brain uptake of ceftriaxone in patients.

Together, at the blood-brain barrier, ABCB1 and ABCG2 restrict brain uptake of substrate drugs and decrease their efficacy, representing a formidable obstacle to the successful therapy of many CNS diseases (Fig. 3C). Thus, understanding ABCB1 and ABCG2 substrate specificities can make the difference between therapeutic failure or success.

#### 3. ABCB1/ABCG2 Substrates

ABCB1 and ABCG2 were first described as mediators of anticancer drug resistance in cancer cells. While ABCB1 was implicated in resistance against daunorubicin, ABCG2 was found to contribute to resistance against mitoxantrone, doxorubicin, and daunorubicin, indicating important roles for both transporters in multidrug resistance (Biedler and Riehm, 1970; Juliano and Ling, 1976; Doyle et al., 1998). de Vries et al. confirmed these findings and showed that Abcb1a/Abcb1b and Abcg2 have overlapping substrate spectra and work together in concert in restricting topotecan brain uptake (de Vries et al., 2007).

Over the past decades, many anticancer drugs have been identified as substrates of either ABCB1, ABCG2, or in many cases both transporters (de Vries et al., 2007; Agarwal and Elmquist, 2012; Traxl et al., 2019). ABCB1/ABCG2 restrict the brain uptake of anticancer drugs and significantly limit their efficacy in the treatment of primary and metastatic brain tumors (Marchetti et al., 2008; Agarwal et al., 2011; de Vries et al., 2012; Taskar et al., 2012; Laramy et al., 2017; de Gooijer et al., 2018a; Sorf et al., 2018). Substrates of ABCB1/ABCG2 are not restricted to a specific class of anticancer drugs but span the entire spectrum of chemotherapeutic compounds. ABCB1/ABCG2 substrates include antibiotics, such as daunorubicin (Juliano and Ling, 1976), alkylating agents like temozolomide (de Gooijer et al., 2018b), microtubule inhibitors including paclitaxel (Kemper et al., 2003, 2004), topoisomerase inhibitors (Marchetti et al., 2008), cell cycle disruptors such as ribociclib (Sorf et al., 2018), and tyrosine kinase inhibitors like lapatinib or sorafenib (Polli et al., 2008, 2009; Agarwal et al., 2011). While many anticancer drugs show promising effects against different brain cancer cell lines in vitro, their efficacy in vivo and in clinical trials has been marginal at best, in large part due to ABCB1/ABCG2-mediated efflux at the blood-brain barrier. Since the seminal work by de Vries et al. in 2007, the overlap in ABCB1 and ABCG2 substrate spectra was expanded from anticancer drugs to include a multitude of other drug classes. ABCB1 significantly restricts brain uptake of some antiseizure drugs and limits their efficacy in the treatment of epilepsy (Cox et al., 2001; van Vliet et al., 2006; Tang et al., 2017). Other drugs that are ABCB1/ABCG2 substrates include HIV protease inhibitors (Kim et al., 1998a,b; Lee et al., 1998), the dopamine hydroxylase inhibitor etamicastat (Bicker et al., 2018), riluzole, one of the few Food and Drug Administration (FDA)-approved drugs for amyotrophic lateral sclerosis (ALS) therapy (Jablonski et al., 2014), and a myriad of drugs including opioids (Letrent et al., 1999; Dagenais et al., 2004; Bauer et al., 2006; Sharma and Ali, 2006; Hassan et al., 2007; Yousif et al., 2008, 2012; Chaves et al., 2016; Schaefer et al., 2018). For example, since oxycodone, morphine, and methadone are weak Abcb1a substrates, they can cross the blood-brain barrier, resulting in substantial brain uptake and CNS activity (Gibbs et al., 2018). On the other hand, active efflux of opioids at the blood-brain barrier has been exploited to develop peripherally active opioids for the treatment of diarrhea. Take loperamide as an example, which is a good Abcb1a/b substrate and therefore does not easily enter the brain (Watari et al., 2019). Loperamide has a four times higher Abcb1a-mediated transport rate compared with methadone, which significantly restricts loperamide brain uptake (Gibbs et al., 2018).

Taken together, ABCB1 and ABCG2 have largely overlapping substrate spectra that comprise a wide range of compounds including anticancer drugs, antiseizure drugs, HIV protease inhibitors, opioids, and a large number of other therapeutically used drugs. The consequence of this overlap in substrates is that both transporters compensate for each other. In other words, drugs directed to the brain have to overcome not one but two transporters—the ABCB1/ABCG2 drug efflux system.

## II. Overcoming The ABCB1/ABCG2 Drug Efflux System

The blood-brain barrier is a challenge in the treatment of many CNS diseases. Over the decades, multiple strategies to overcome the blood-brain barrier have been developed with the goal of improving drug therapy for CNS disorders. These strategies can largely be divided into transporter-independent and transporter-dependent strategies.

Transporter-independent strategies to overcome the barrier include blood-brain barrier disruption with focused ultrasound (Hynynen et al., 2001; Burgess et al., 2011; Mainprize et al., 2019), hyperosmotic solutions (Neuwelt et al., 1986; Doolittle et al., 2000; Angelov et al., 2009; Chakraborty et al., 2016; Lesniak et al., 2019), transport vehicles that target receptor-mediated transcytosis (Pardridge, 2001; Kariolis et al., 2020; Ullman et al., 2020), direct drug delivery via intraparenchymal infusion, waver implantation (Valtonen et al., 1997) or convection-enhanced delivery (Laske et al., 1997; Lidar et al., 2004), intranasal delivery (Thorne et al., 1995; Frey, 1997, 2001), or the use of liposomes and nanoparticles (Huwyler et al., 1996; Ulbrich et al., 2009; Fan et al., 2018). Currently, only intraarterial injection of hyperosmotic mannitol and implantable drug wavers are FDA-approved therapeutics (National Cancer Institute, 2022). Other transporter-independent strategies exist (Doolittle et al., 2000; Hynynen et al., 2001; Westphal et al., 2003; Duntze et al., 2013; Mainprize et al., 2019; Kariolis et al., 2020; Ullman et al., 2020).

**Fig. 4:**
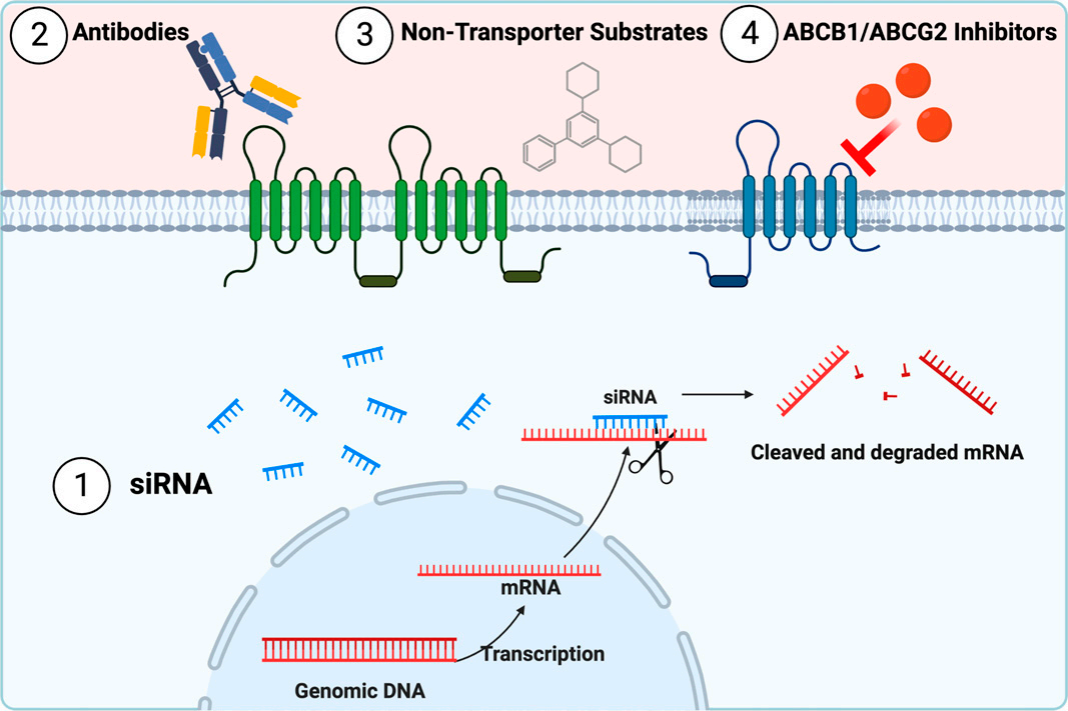
Transporter-dependent strategies to overcome ABCB1 and ABCG2 drug efflux. Transporter-dependent strategies focus on inhibiting and overcoming ABCB1- and ABCG2-mediated drug efflux by using (1) siRNA, (2) antibodies, (3) nontransporter substrates, or (4) transporter inhibitors. Created with BioRender.com.

In the following section, we discuss advantages and disadvantages of the main transporter-dependent strategies. Transporter-dependent strategies (Fig. 4) focus on inhibiting and overcoming ABCB1- and ABCG2-mediated drug efflux using small interfering (siRNA), antibodies, nontransporter, or transporter inhibitors.

### A. Small Interfering RNA

In vitro, siRNA reduce *ABCB1*/*ABCG2* mRNA and ABCB1/ABCG2 protein expression in drug-resistant cancer cells such as U87 glioblastoma cells or hepatocellular carcinoma cells (Fisher et al., 2007; Zhao et al., 2008; Li et al., 2012). ABCB1/ABCG2 knockdown also decreases transporter function, increases the accumulation of rhodamine 123, and enhances the cytotoxicity of doxorubicin (Rittierodt et al., 2004; Fisher et al., 2007; Zhao et al., 2008; Li et al., 2012). However, synthetic siRNAs have not yet been tested in vivo.

While superficially similar to siRNA, microRNAs (miRNA) have several specific differences. Both are endogenous, small noncoding RNAs that act as a recognition sequence to permit the RNA-induced silencing complex to bind target mRNAs. However, siRNAs silence genes by cleaving mRNA before translation, while miRNAs function to silence the translation apparatus. In addition, siRNA targeting relies on (near) 100% complementarity, whereas miRNA binding requirements are less stringent. The stem-loop structures that give rise to miRNAs are also shorter than the long double-stranded RNA that gives rise to siRNA (Mack, 2007; Qureshi et al., 2014).

miRNAs target the 3′-untranslated region (3′-UTR) of an mRNA. Binding of the 3′-UTR prevents the assembly of the translational complex and decreases the expression of the target protein (Ambros, 2004; Bartel, 2004). However, in some instances, miRNAs target other regions of an mRNA, including the 5′-UTR and the protein-coding sequence, and miRNA activity upregulates translation of some targets (Long et al., 2019) Several groups have identified differentially expressed miRNAs in tumor cells and at the blood-tumor barrier in samples from glioblastoma patients. For example, expression of miR-145 is decreased in tumor samples from glioblastoma patients. Transfecting U87 glioblastoma cells with synthetic miR-145 decreases both ABCB1 and ABCG2 protein levels, which in return increases sunitinib cytotoxicity (Liu et al., 2015). Another miRNA, miR-4539, altered expression of ABCB1 in T98G glioblastoma cells and aligned strongly with the 3′-UTR mRNA sequence of *ABCB1*. Cotreatment of cells with doxorubicin and miR-4539 increased toxicity by at least 40%, depending on miRNA dose (Medarova et al., 2020).

While these in vitro data seem promising, only a few in vivo studies have been conducted in animal disease models. Li et al. showed that miR-378 increases the treatment response in orthotopic glioblastoma mouse models in vivo (Li et al., 2018). Deng et al. found that miR-146a-5p expression is lower in the brains of rats after status epilepticus compared with control rats (Deng et al., 2019). Downregulation of miR146a-5p increased Abcb1 protein levels at the blood-brain barrier of rats with status epilepticus. Additionally, injecting miR-146a-5p into the hippocampus of rats with status epilepticus decreased *Abcb1* mRNA and protein expression (Deng et al., 2019). However, these authors did not evaluate treatment response. To fully evaluate the impact of siRNA and miRNA approaches on drug brain delivery and efficacy, further studies in animal models are necessary.

### B. Antibodies

Several anti-ABCB1 antibodies have been tested both in vitro and in vivo. The antibodies MRK16 and MRK17 inhibited Abcb1-mediated efflux in vitro and in animal tumor models and increased doxorubicin efficacy (Broxterman et al., 1988; Tsuruo et al., 1989; Mechetner and Roninson, 1992). MRK16 also increased efficacy of other anticancer drugs in ABCB1-overexpressing cells but had no effect on the parent cells (Hamada and Tsuruo, 1986; Pearson et al., 1991). Other antibodies, such as MRK17 and UIC2, had cytotoxic effects themselves, possibly through antibody-dependent cytotoxicity (Hamada and Tsuruo, 1986; Mechetner and Roninson, 1992). While these initial studies were promising, this strategy has not been further developed since the mid-1990s.

### C. Nontransporter Substrates

Another approach has been to develop compounds that are pharmacologically active in the CNS but not substrates for blood-brain barrier efflux transporters. Among these nontransporter substrates are the epidermal growth factor receptor (EGFR) inhibitors buparlisib and avitinib (Heffron et al., 2016a,b; Sio et al., 2014; Wu et al., 2017; de Gooijer et al., 2018c; Wang et al., 2018). However, both drugs are associated with significant adverse effects (Borson-Chazot et al., 2018; Di Leo et al., 2018). The PI3K/Akt/mTOR inhibitor GDC-0084 had promising brain distribution and efficacy in preclinical models (Heffron, 2016; Salphati et al., 2016) but was ineffective in a recent phase 1 clinical trial in patients with recurrent glioblastoma (Wen et al., 2020). Thus, developing CNS drugs that are neither an ABCB1 nor an ABCG2 substrate is challenging.

### D. ABCB1/ABCG2 Inhibitors

In the past decades, most research efforts in the drug efflux transporter field have been spent on developing inhibitors for ABCB1, ABCG2, or dual inhibitors for both transporters. Tsuruo et al. were the first to discover that the calcium channel blocker verapamil overcomes ABCB1-mediated resistance against vinca alkaloids (Tsuruo et al., 1981; Broxterman et al., 1988). However, due to its primary effect on the cardiovascular system, verapamil is associated with cardiovascular toxicity (Pennock et al., 1991). Similarly, cyclosporin A, another promising first-generation ABCB1 inhibitor, is associated with immunosuppression, nephrotoxicity, and hemodynamic adverse events (Mechetner and Roninson, 1992; Tsuji et al., 1993; Desrayaud et al., 1997). While both verapamil and cyclosporin A enhanced brain delivery of several drugs in animal brain cancer and epilepsy models (Tatsuta et al., 1992; Chikhale et al., 1995; Drion et al., 1996; Cox et al., 2001), responses were small due to their low ABCB1 binding affinity and competitive transporter inhibition that was easily overcome (Cisternino et al., 2001; Kemper et al., 2003; Thomas and Coley, 2003). Today, first-generation ABCB1 inhibitors are used as positron emission tomography tracers to test the efficacy of newly developed transporter inhibitors in humans (Hendrikse et al., 1999; Bankstahl et al., 2008; Bauer et al., 2017).

Second-generation ABCB1 inhibitors, like valspodar (PSC833), were developed with increased potency and reduced off-target effects and toxicity (Boesch et al., 1991; Friche et al., 1992; List, 1996; Tidefelt et al., 2000; Thomas and Coley, 2003). Data from in vivo studies in mice show that valspodar increased brain levels of several Abcb1 substrates without affecting their plasma pharmacokinetics (Drion et al., 1996; Desrayaud et al., 1997; Mayer et al., 1997; Cisternino et al., 2001; Kemper et al., 2003; Hubensack et al., 2008). Fellner and colleagues (2002) demonstrated that valspodar given in combination with paclitaxel reduced tumor volume by 90% in a mouse glioblastoma model and concluded that Abcb1 inhibition would potentially allow anticancer drugs to reach a tumor in the brain (Fellner et al., 2002). However, second-generation ABCB1 inhibitors inhibit several other ABC transporters due to their low selectivity and are highly bound to plasma proteins (Simon et al., 1998; Thomas and Coley, 2003). Moreover, many second-generation ABCB1 inhibitors are metabolized by CYP 450 enzymes, resulting in drug-drug interactions (Wandel et al., 1999; O’Byrne et al., 2001; Kemper et al., 2003).

Third-generation ABCB1 inhibitors such as tariquidar (XR9576) and elacridar (GF120918) are highly specific and lack CYP 450 enzyme interactions (Thomas and Coley, 2003). These inhibitors induce long-lasting, dose-dependent Abcb1 inhibition without causing adverse effects in mice (Stewart et al., 2000; Abraham et al., 2001, 2009; Cisternino et al., 2001; Ferry et al., 2001; Thomas et al., 2001; Dorner et al., 2009). Both elacridar and tariquidar increase the delivery of drugs into the brain, including anticancer drugs, opioids, and HIV protease inhibitors (Letrent et al., 1999; Edwards et al., 2002; Kemper et al., 2003; Walker et al., 2004; Pusztai et al., 2005; Choo et al., 2006; van Vliet et al., 2006; Fox and Bates, 2007; Bankstahl et al., 2008; Hubensack et al., 2008; Kurnik et al., 2008; Chen et al., 2009; Lagas et al., 2009, 2010; Agarwal et al., 2011; Hendrikx et al., 2014; Traxl et al., 2015; Mittapalli et al., 2016). Tariquidar and elacridar inhibit the ATPase activity of human ABCB1 and mouse Abcb1 and were initially thought to not interact with the substrate binding site (Martin et al., 1999; Cisternino et al., 2001; Mistry et al., 2001; Dorner et al., 2009). However, tariquidar has recently been shown to be both an ABCB1 and ABCG2 inhibitor and substrate (Kannan et al., 2011). Drawbacks of third-generation ABCB1 inhibitors include that they are poorly soluble and have highly variable pharmacokinetics depending on the route of administration (Ward and Azzarano, 2004; Sane et al., 2012; Matzneller et al., 2018). Today, third-generation ABCB1 inhibitors are also used in positron emission tomography imaging to determine brain uptake in the preclinical and clinical setting (Wagner et al., 2009; Bauer et al., 2010; Bauer et al., 2019; Traxl et al., 2019).

Current research efforts are focused on developing extended-release formulations to overcome solubility and pharmacokinetic issues associated with transporter inhibitors (Matzneller et al., 2018). The development of ABCB1/ABCG2 inhibitors is further complicated by species differences in transporter expression and activity levels. These differences become apparent when comparing rodents with humans but also when comparing humans with other higher species, including monkeys or dogs (Roulet et al., 2003; Haritova et al., 2008; Kamiie et al., 2008; Ito et al., 2011; Uchida et al., 2011). Thus, the “ideal” transporter inhibitor is yet to be found.

In summary, none of the described transporter-dependent strategies to overcome the blood-brain barrier in general or ABCB1/ABCG2 specifically were clinically successful, mostly due to low efficacy, high toxicity, and frequent adverse events, especially in combination with standard of care treatment. Therefore, new strategies to overcome ABCB1/ABCG2-mediated efflux at the blood-brain barrier and to improve the treatment of CNS disorders need to be pursued. One such strategy is to pharmacologically target molecules that directly or indirectly regulate the expression and activity of ABCB1 and ABCG2 at the blood-brain barrier (Hartz and Bauer, 2010). In the following section we summarize current knowledge of ABCB1 and ABCG2 regulation and discuss target molecules that could be used to modulate blood-brain barrier transporter expression and activity.

## III. Regulation of ABCB1/ABCG2 at the Blood-Brain Barrier

Three main pathways regulate ABCB1 and ABCG2 at the blood-brain barrier: (1) nuclear receptors, (2) inflammatory and oxidative stress signaling, and (3) receptor tyrosine kinase/growth factor signaling. In the following sections we describe these pathways in detail and highlight their clinical significance where appropriate. We also summarize other signaling mechanisms involved in ABCB1/ABCG2 regulation and briefly describe their clinical relevance.

### A. Nuclear Receptors

Nuclear receptors are critical transcription factors. By binding directly to DNA and inducing or inhibiting the transcription of target genes, nuclear receptors regulate important cellular functions in development, homeostasis, and metabolism (Evans, 1988; Olefsky, 2001). Ligands of nuclear receptors are classified as hormones, vitamins, or xenobiotic endocrine disruptors (Overington et al., 2006). After activation by their respective ligands, nuclear receptors form homo- or heterodimers with heat shock protein or retinoid X receptor (RXR) that bind to specific response elements in the promotor regions of their target genes (Klinge et al., 1997; Linja et al., 2004; Amoutzias et al., 2007). For *ABCB1* and *ABCG2,* the response elements for several nuclear receptors are located in their respective proximal promotor (Nakanishi and Ross, 2012). Therefore, activation of nuclear receptors regulates the transcription of *ABCB1* and *ABCG2* changing transporter expression and activity at the blood-brain barrier but also in other barrier organs, such as placenta, testes, intestine, liver, and kidney (Rigalli et al., 2019b). With a few exceptions, this process involves transcription and translation and is, therefore, relatively slow (Miller and Cannon, 2014).

#### 1. Corticoid Receptors

In 1992, Loffreda et al. were the first to detect nuclear receptors at the blood-brain barrier. (Loffreda et al., 1992). These researchers found mineralocorticoid receptor (MR) and glucocorticoid receptor (GR) mRNA in isolated rat brain capillaries. Stimulating mineralocorticoid and GR with dexamethasone increases *Abcb1* and *Abcg2* expression in vitro in primary rat brain capillary endothelial cells and in vivo at the mouse blood-brain barrier (Narang et al., 2008; Petropoulos et al., 2010; Chan et al., 2013; Miller, 2015; Yasuda et al., 2015; Chaves et al., 2017). This effect was dose-dependent and reversible and could be inhibited with GR antagonists (Narang et al., 2008).

**Fig. 5:**
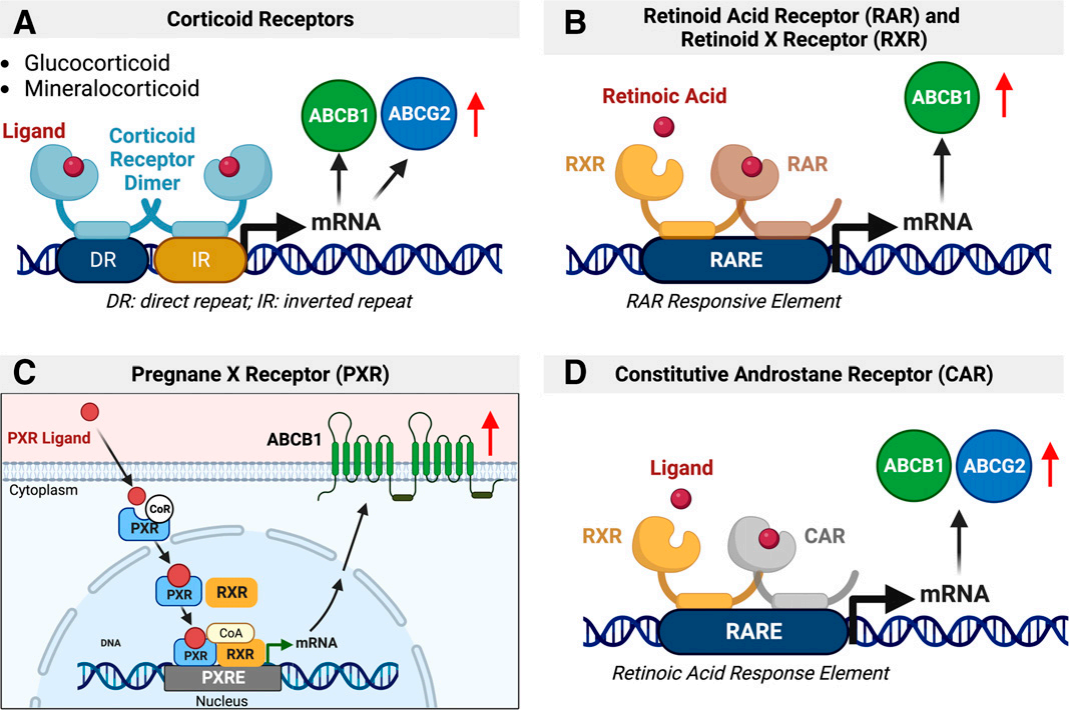
Regulation of ABCB1 and ABCG2 via corticoid receptors RAR/RXR, PXR, and CAR. (A) Upon ligand binding, the corticoid receptor dimer binds to the direct repeat and inverted repeat region of the target gene to increase ABCB1 and ABCG2 mRNA expression levels. (B) Upon ligand binding, RAR and RXR form a heterodimer that binds and activates the RAR response element (RARE), which increases ABCB1 expression. (C) A PXR ligand binds to inactivated PXR in the cytoplasm. Ligand binding then triggers conformational change of PXR during which the corepressor dissociates. Activated PXR translocates into the nucleus and heterodimerizes with retinoic X receptor *α* (RXR*α*). The complex PXR-RXR*α* together with its coactivators binds to the xenobiotic response element in the promotor region on ABCB1. This results in increased transcription of the gene and protein expression. (D) CAR forms a heterodimer with RXR that binds to RARE, which leads to an increase in ABCB1 and ABCG2 levels. Created with BioRender.com.

In general, corticoid receptors are activated by endogenous hormones such as glucocorticoids as well as exogenous xenobiotics (Fig. 5A). Upon activation, the receptor translocates from the cytoplasm to the nucleus, where it binds to the response element of its target genes, resulting in transcription (Miller, 2010). Corticoid signaling is critical during blood-brain barrier development. Activation of maternal GR during development induces early *Abcb1* expression in brain capillaries isolated from Guinea pig fetuses at different developmental stages (Iqbal et al., 2016).

Glucocorticoids are often used to prevent edema in patients with brain tumors. However, glucocorticoid activation of GR upregulates *ABCB1*/*ABCG2* at the blood-brain barrier. Increased efflux transporter expression and activity then further restricts brain uptake of anticancer drugs, limiting their efficacy in the treatment of brain tumors (Petropoulos et al., 2010).

#### 2. Retinoid Acid Receptor and Retinoid X Receptor

In 1997, El Hafny et al. showed that retinoic acid increases *Abcb1* expression and ABCB1 activity in a rat brain capillary endothelial cell line in a concentration-dependent manner. Retinoic acid binds to retinoid acid receptor (RAR) and induces the formation of heterodimers with RXR (Fig. 5B). The RAR-RXR heterodimer activates the retinoic acid response element in the *Abcb1* promotor resulting in transporter upregulation (El Hafny et al., 1997). A similar process involves several other RAR ligands (Xu et al., 2005; Sarkadi et al., 2006; Chan et al., 2013).

#### 3. Pregnane X Receptor

In 1998, Kliewer and colleagues discovered pregnane X receptor (PXR) (Kliewer et al., 1998). PXR functions as a xenobiotic sensor, and its activation increases levels of proteins involved in detoxification and xenobiotic clearance (Kliewer et al., 1998). Upon activation, PXR forms heterodimers with RXR (Bauer et al., 2005) or other orphan nuclear receptors (Xu et al., 2005) and binds to its response element in the promotor region of its target genes (Fig. 5C; Song et al., 2004; Miller et al., 2008). In 2001, Synold et al. (2001) showed that PXR regulates ABCB1 protein levels. We found that PXR is expressed in isolated rat brain capillaries and first reported that PXR activation upregulates rodent Abcb1 protein levels and transport activity at the blood-brain barrier (Bauer et al., 2004, 2006; Hartz and Bauer, 2010). Other groups later confirmed our findings and also showed that PXR activation increases Abcb1 *and* Abcg2 protein levels and activity at the blood-brain barrier in rodents (Chan et al., 2011; Yasuda et al., 2015; Chaves et al., 2017).

Many drugs that are ABCB1 and ABCG2 substrates increase their own efflux at the blood-brain barrier through PXR-mediated upregulation. For example, antiretroviral drugs, antiseizure drugs, and several other drugs, including rifampicin and hyperforin, are PXR agonists. These drugs induce *ABCB1*/*ABCG2* expression at the blood-brain barrier by activating PXR and, thus, restrict their own brain uptake and efficacy (Chan et al., 2011; Potschka, 2012; Chan et al., 2013).

#### 4. Constitutive Androstane Receptor

Upon activation, constitutive androstane receptor (CAR) forms heterodimers with RXR, which bind to the retinoic acid response element in the promotor sequence of target genes (Xu et al., 2005; Sarkadi et al., 2006; Fig. 5D). Both *Abcb1* and *Abcg2* are among those target genes. In this regard, xenobiotics and drugs, such as phenobarbital, increase the expression of both *Abcb1* and *Abcg2* and their accompanying proteins’ activity in isolated brain capillaries from mice and rats (Wang et al., 2010; Yasuda et al., 2015) This also occurs in hCMEC/D3 cells, a human brain microvascular endothelial cell line (Chan et al., 2011). *ABCB1* mRNA and ABCB1 protein levels increased after exposing hCMEC/D3 cells to the CAR ligand 6-(4-chlorophenyl)imidazo[2,1-b][1,3]thiazole-5-carbaldehyde O-(3,4-dichlorobenzyl)oxime. This upregulation was inhibited by coexposing the cells to 6-(4-chlorophenyl)imidazo[2,1-b][1,3]thiazole-5-carbaldehyde O-(3,4-dichlorobenzyl)oxime and the CAR inhibitor meclizine (Chan et al., 2011). In a follow-up study, Chan et al. demonstrated that the antiretroviral drugs abacavir, efavirenz, and nevirapine are CAR ligands and upregulate *ABCB1* in hCMEC/D3 cells (Chan et al., 2013).

Acetaminophen is a common over-the-counter pain and fever-relieving agent. High doses of acetaminophen activate CAR, which increases *Abcb1* mRNA levels and accompanying protein activity in isolated rat brain capillaries (Slosky et al., 2013). In addition, five FDA-approved drugs were identified that facilitate CAR transport into the nucleus, including the antihypertensive drug telmisartan (Lynch et al., 2013). These drugs could potentially affect transporters at the blood-brain barrier.

#### 5. Peroxisome Proliferator-Activated Receptor

**Fig. 6:**
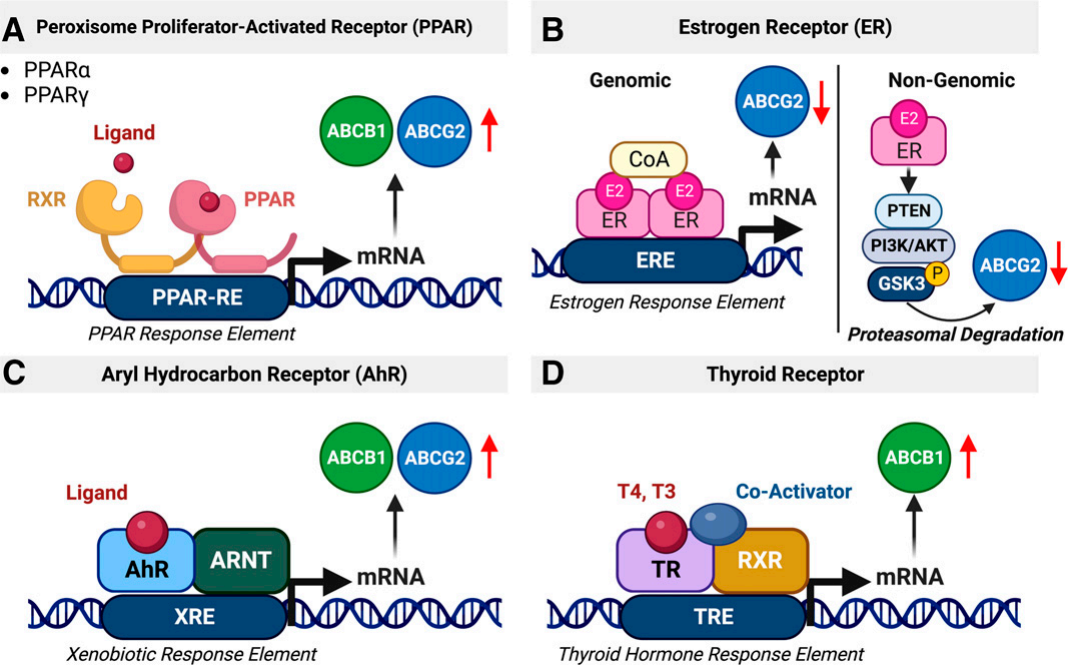
Regulation of ABCB1 and ABCG2 via the nuclear receptors PPAR, ER, AhR, and thyroid hormone receptor. (A) PPAR forms a heterodimer with RXR that binds to and activates the PPAR response element, which leads to increased ABCB1 and ABCG2 levels. (B) Genomic regulation of ABCG2 is driven by the estrogen receptor that binds to the estrogen response element in the ABCG2 promotor region. In addition, ABCG2 is also regulated via rapid, nongenomic ER signaling involving PTEN/PI3K/Akt/GSK3. (C) AhR translocates into the nucleus and dimerizes with the aryl hydrocarbon receptor nuclear translocator resulting in the regulation of its target genes, including ABCB1 and ABCG2. (D) The thyroid receptor forms a complex with RXR and coactivators. This complex binds to the thyroid hormone response element and activates transcription of ABCB1. Created with BioRender.com.

Three types of peroxisome proliferator-activated receptors (PPAR*α*, PPAR*β*, PPAR*γ*) have been identified, but only PPAR*α* and PPAR*γ* are involved in ABC transporter regulation (Xu et al., 2005). Clofibrate, linoleic acid, and other PPAR*α* agonists increase expression levels of *Abcb1* and *Abcg2* mRNAs and accompanying proteins as well as transporter activity in isolated mouse brain capillaries and in hCMEC/D3 cells (Hoque et al., 2012; Chan et al., 2013; Hoque et al., 2015; More et al., 2017). After heterodimerizing with RXR, the PPAR*γ*/RXR complex binds to the PPAR response element upstream of the *Abcg2* promotor, which induces drug resistance in cancer cells (Nakanishi and Ross, 2012; Fig. 6A). PPAR*γ* also regulates *ABCB1* and *ABCG2* in human glioblastoma cell lines in vitro (Szatmari et al., 2006; Han et al., 2015). Cannon et al. (2020) showed that ammonium 2,3,3,3-tetrafluoro-2-(heptafluoropropoxy) propanoate, a chemical precursor used in the production of Teflon, rapidly inhibits *Abcb1* transport activity in isolated rat brain capillaries and that this inhibition is dependent on PPAR*γ* activity.

Fibrates, a class of drugs used treat hypercholesterolemia, are PPAR*α* agonists. Clofibrate upregulates *Abcb1*/*Abcg2* mRNA and associated protein levels as well as efflux transporter activity in isolated rat brain capillaries and in hCMEC/D3 cells in vitro (Hoque et al., 2015; More et al., 2017). The thiazolidinediones are a class PPAR*γ* agonists approved for treatment of type II diabetes and include pioglitazone, rosiglitazone, and lobeglitazone. Of these, pioglitazone increases docosahexaenoic acid trafficking into the brain (Low et al., 2020), crosses the blood-brain barrier, and reduces tumor growth in a human xenograft model (Grommes et al., 2013). Rosiglitazone, on the other hand, appears to reinforce the integrity of the blood-brain barrier (Sivandzade and Cucullo, 2019; Zhao et al., 2019).

#### 6. Estrogen Receptor

Estrogen receptors (ERs) are hormone-activated nuclear receptors (ER*α* and ER*β*) or G-protein coupled membrane receptors (GPR30, ER-X, and G_q_-mER). Estrogen binding to these receptors triggers either a rapid (minutes) response through nongenomic pathways or a slow response (hours-days) through genomic signaling pathways (Fig. 6B).

In 2002, Imai et al. (2002) showed that 17*β*-estradiol enhances the cytotoxicity of several anticancer drugs in vitro by decreasing expression of *ABCG2* in human leukemia cells (Imai et al., 2002). The estrogen response element was detected in the *ABCG2* promotor region (Ee et al., 2004). In addition to the genomic regulation of *ABCG2,* Imai et al. (2005) also discovered that 17*β*-estradiol activation of ER*α* increases topotecan cytotoxicity via a nongenomic pathway through posttranscriptional regulation of *ABCG2* in human breast cancer cells (Imai et al., 2005). In 2010, we showed that estrogen signaling regulates *Abcg2* at the blood-brain barrier (Hartz et al., 2010a,b). We found that 17*β*-estradiol (E2) decreased Abcg2 activity within minutes and this effect did not involve transcription, translation, or proteasomal degradation, indicating a nongenomic mechanism (Hartz et al., 2010b). Experiments with ER*α* and ER*β* knockout mice showed that rapid loss of Abcg2 activity was due to E2 signaling through both receptors. In a follow-up study we demonstrated that 6-hour E2 exposure of isolated brain capillaries resulted in a loss of Abcg2 activity that was accompanied by reduced Abcg2 protein expression levels. Altogether, we found that the signaling process responsible for these effects in isolated rat brain capillaries involved E2 signaling through ER*β*, which inhibits the PTEN/PI3K/Akt/GSK3 pathway leading to Abcg2 proteasomal degradation (Hartz et al., 2010b). Thus, E2 acting through either ER can signal an initial loss of Abcg2 transport activity, but only signaling through ER*β* mediates reduced ABCG2 protein expression and activity levels.

Another estrogenic compound, the synthetic xenoestrogen bisphenol A, is a common component of plastic products that also activates ERs. Bisphenol A decreased Abcg2 protein and activity levels in isolated rat brain capillaries via an ER*α*-dependent genomic pathway (Nickel and Mahringer, 2014). Specifically, upon bisphenol A-mediated activation, ER*α* binds to the estrogen response element in the *Abcg2* promotor where it acts as a negative regulator resulting in a slow decrease in *Abcg2* expression and activity levels in isolated mouse brain capillaries (Zhang et al., 2010; Shin et al., 2018). Phytoestrogens from soybeans also induce *ABCG2* expression and protein activity through a genomic signaling pathway in breast cancer cell lines (Rigalli et al., 2019a,b). However, this particular pathway has not yet been identified at the blood-brain barrier. A similar ER*β*-dependent, nongenomic pathway for ABCB1 that is activated by androstanes also exists (Zuloaga et al., 2012). Further information on ER-dependent, nongenomic *ABCB1* and *ABCG2* regulation is in Section C.3.

#### 7. Aryl Hydrocarbon Receptor

The aryl hydrocarbon receptor (AhR) does not belong to the family of 48 known human nuclear receptors but is a member of the basic Helix-Loop-Helix-Period/ARNT/Single-minded family of dimerizing transcription factors. Similar to xenobiotic-sensing nuclear receptors, after binding and activation by aromatic aryl hydrocarbons, from which its name derives, AhR translocates into the nucleus and dimerizes with the aryl hydrocarbon receptor nuclear translocator (Xu et al., 2005; Fig. 6C) resulting in the regulation of its target genes, including transporters. AhR is highly expressed in hCMEC/D3 cells (Dauchy et al., 2008, 2009) and increases *Abcb1* and *Abcg2* mRNA expression levels and activity levels of the respective proteins in several tissues, including the blood-brain barrier of mice and rats (Klaassen and Slitt, 2005; Campos et al., 2012; Nakanishi and Ross, 2012; Chan et al., 2013; Le Vee et al., 2015; Chaves et al., 2017). AhR inhibition with ethanol decreases *Abcb1* and *Abcg2* mRNA expression and associated protein levels at the rat blood-brain barrier (Hammad et al., 2019), but other AhR signaling in brain endothelial cells is unknown.

#### 8. Thyroid Receptors

Thyroid hormone signaling regulates processes including growth, development, and metabolism. The main thyroid hormones are thyroxin (T4) and 3,3,3′-triiodo-L-thyronine, which enter the brain by crossing the blood-brain barrier. The role of thyroid hormones in the regulation of ABCB1 and ABCG2 at the blood-brain barrier, however, is not well investigated and limited to few studies. In this regard, Saljé et al. (2012) treated rats with T4 (9 *μ*g/kg for 9 days) and showed upregulation of *Abcb1* protein expression in brain and liver tissue (Fig. 6D). Kassem and colleagues (2007) found that *ABCB1* regulates T4 levels in the CSF by facilitating T4 transport between the choroid plexus, the brain, and the CSF. However, thyroid regulation of blood-brain barrier ABCB1 and ABCG2 remains largely unexplored.

#### 9. Other Nuclear Receptors

Several other nuclear receptors such as Farnesoid X receptor (FXR), liver X receptor (LXR), and vitamin D receptor (VDR) have been implicated in transporter regulation at the blood-brain barrier. Thus far, however, they have not been studied in detail, and little is known about their role in transporter regulation at the blood-brain barrier. For example, the FXR ligand chenodeoxycholic acid upregulates the efflux transporter Abcc2 in isolated rat brain capillaries, indicating that FXR could be involved in the regulation of its target genes at the blood-brain barrier (Bauer et al., 2008a). Further, the VDR regulates protein expression levels of both ABCB1 and ABCG2 (Sarkadi et al., 2006; Chan et al., 2013; Chaves et al., 2017). In contrast, the LXR regulates *Abca1* mRNA levels in an immortalized rat brain capillary endothelial cell line (TR-BBB13) but has no effect on *Abcg2* mRNA levels (Akanuma et al., 2008). More studies are needed to understand the role FXR, LXR, and VDR play in transporter regulation at the blood-brain barrier.

### B. Inflammatory and Oxidative Stress Signaling

#### 1. Inflammation

The brain is not immune-privileged, as originally anticipated, and immune cells do cross the blood-brain barrier and enter the brain (Engelhardt et al., 2017). The barrier itself contributes to inflammation, and brain capillary endothelial cells respond to inflammatory stimuli and release cytokines. Neuroinflammation is common among all CNS diseases including epilepsy (Choi and Koh, 2008; Alyu and Dikmen, 2017; Rana and Musto, 2018), brain tumors (Jiang et al., 2017; Couto et al., 2019), and Alzheimer’s disease (Akiyama et al., 2000; Wyss-Coray and Rogers, 2012; Mosher and Wyss-Coray, 2014; Lai et al., 2017). Neuroinflammatory signaling is driven by cytokines and oxidative stress, both of which are implicated in the regulation of blood-brain barrier transporters through activating different signaling pathways. In the following sections, we discuss four key regulators of ABC transporters at the blood-brain barrier: (1) nuclear factor kappa-light-chain-enhancer of activated B cells (NF-*κ*B), (2) tumor necrosis factor *α* (TNF*α*), (3) prostaglandins, and (4) cytokines.

##### a. Nuclear factor kappa-light-chain-enhancer of activated B cells

NF-*κ*B is a rapidly acting, primary transcription factor that is constitutively expressed in the cytoplasm of all cells (Jacobs and Harrison, 1998). In its inactive state, NF-*κ*B is bound to the inhibitor of *κ*B, which prevents translocation into the nucleus (Jacobs and Harrison, 1998). Upon activation by infectious and inflammatory stimuli or through cell surface receptors, inhibitor of *κ*B is ubiquitinated and degraded, which releases NF-*κ*B, allowing translocation into the nucleus. There, NF-*κ*B binds to the promoters of its target genes and stimulates transcription (Deptala et al., 1998; Gilmore, 2006). Among NF-*κ*B target genes are proinflammatory cytokines as well as markers of cell survival and proliferation (Renard et al., 1997; Basu et al., 1998; Deptala et al., 1998; Chandel et al., 2000; Qin et al., 2005; Fitzgerald et al., 2007).

In 2005, Dixit et al. showed that interferon *γ* stimulates *ABCB1* expression and ABCB1 activity in human intestinal cells in vitro via NO synthase and NF-*κ*B (Dixit et al., 2005). Pan et al. discovered a similar pathway showing that lipopolysaccharide-induced inflammation increases *Abcb1* mRNA expression levels at the blood-brain barrier of wild-type but not NF-*κ*B knockout mice, indicating an important role for NF-*κ*B in *Abcb1* regulation (Pan et al., 2010). Since then, several groups have shown that NF-*κ*B activation by inflammatory stimuli or cellular stress increases the expression of *Abcb1* and associated protein activity at the rodent blood-brain barrier in vivo and in vitro (Bauer et al., 2007; Pan et al., 2010; Ronaldson et al., 2010; Zhang et al., 2014). In contrast, stimuli that inhibit NF-*κ*B signaling decrease *Abcb1* and *Abcg2* mRNA expression and associated protein activity levels at the blood-brain barrier. For example, in cultured rat microvessel endothelial cells in vitro, insulin inhibits NF-*κ*B through the insulin receptor, which decreases both *Abcb1* and *Abcg2* mRNA expression and protein activity levels (Liu et al., 2009; Liu et al., 2011). Additionally, in vivo experiments in diabetic rats showed the opposite effect: increased ABC transporter expression and activity at the blood-brain barrier due to decreased insulin plasma levels (Maeng et al., 2007). Thus, NF-*κ*B is a key transcription factor that regulates both Abcb1 and Abcg2 at the blood-brain barrier.

Multiple drug candidates for repurposing to regulate NF-*κ*B include clemastine, topotecan, bortezomib, and dexamethasone (Roberti et al., 2022) and are known to affect ABC transporters (Hartz et al., 2016). Other drugs, like methamphetamine, weaken the blood-brain barrier by inhibiting NF-*κ*B (Coelho-Santos et al., 2015), and another drug of abuse, mephedrone, activates NF-*κ*B and increases blood-brain barrier permeability (Buzhdygan et al., 2021). At this point, it is unclear if these compounds affect ABC transporters.

##### b. Wnt/β-catenin signaling

Wnt/*β*-catenin signaling is part of several inflammatory signaling cascades. During canonical Wnt signaling, *β*-catenin is degraded by a so-called “destruction complex” formed by GSK3*β*, APC, and axin. Upon activation, Wnt binds to the Frizzled receptor, which recruits axin and inhibits GSK3Β. Consequently, the destruction complex cannot assemble, and *β*-catenin accumulates in the cytosol. After translocation into the nucleus, *β*-catenin acts as transcription factor and induces transcription of genes involved in cell proliferation and survival (Atlasi et al., 2014). At the blood-brain barrier, *β*-catenin regulates the transcription of *Abcb1* and *Abcg2. β*-catenin leads to increased transporter expression and activity levels at the blood-brain barrier in vitro in hCMEC/D3 cells and in mice and rats in vivo (Lim et al., 2008, 2009; Harati et al., 2013; Paolinelli et al., 2013; Strazielle and Ghersi-Egea, 2015; Laksitorini et al., 2019).

##### c. Tumor necrosis factor alpha

**Fig. 7:**
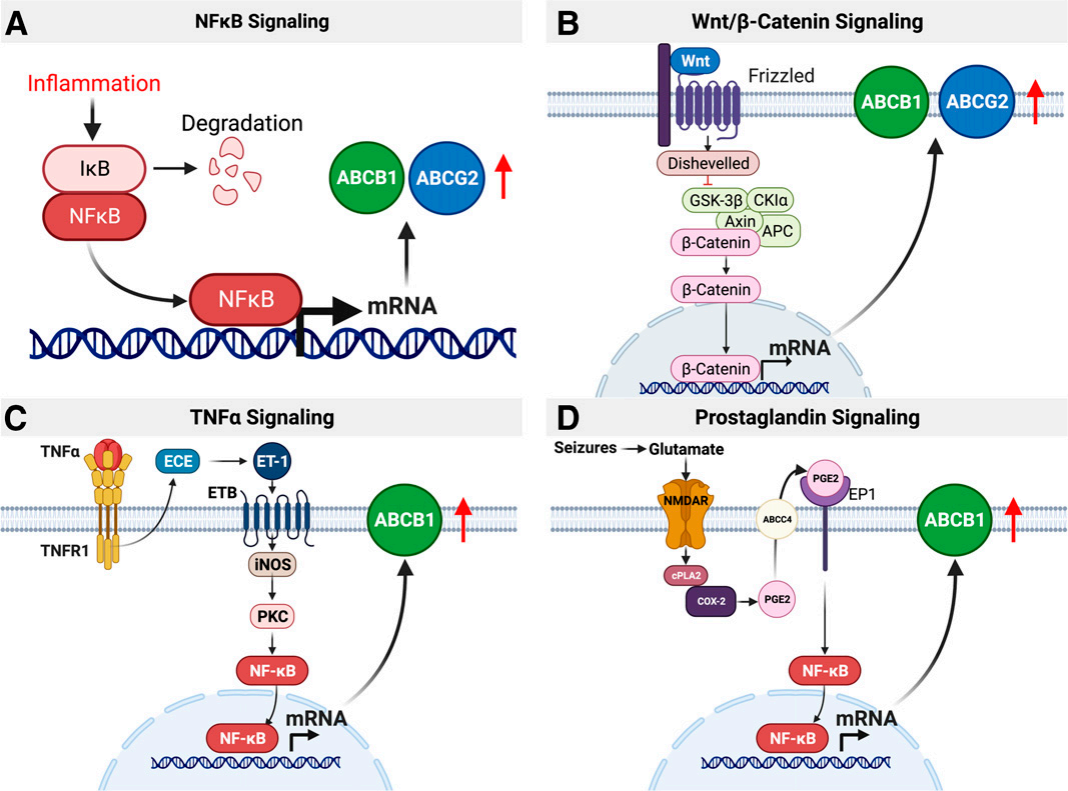
Inflammatory and oxidative stress signaling. (A) NFkB, a primary transcription factor, is activated by infectious and inflammatory stimuli. NF-*κ*B binds to the promoters of its target genes and stimulates transcription. Both ABCB1 and ABCG2 are regulated via NFkB signaling at the blood-brain barrier. (B) Upon activation, Wnt binds to the Frizzled receptor, which recruits axin and inhibits GSK3Β. Consequently, the destruction complex cannot assemble, and *β*-catenin accumulates in the cytosol. After translocation into the nucleus, *β*-catenin acts as transcription factor and induces transcription of both ABCB1 and ABCG2. (C) In isolated brain capillaries, TNF*α* signals through TNF receptor 1 activating the endothelin converting enzyme, which, in turn, leads to the production of endothelin 1, which signals through the endothelin receptor B to activate the inducible nitric oxide synthase. NO stimulates protein kinase C, which leads to the activation of NF-*κ*B, which upregulates ABCB1 protein expression and transport activity. (D) Seizure-induced glutamate release activates NMDAR- cytosolic phospholipase A2-COX-2 signaling that leads to the generation of prostaglandin E2 (PGE2) by the microsomal prostaglandin synthase. PGE2 activates the prostaglandin EP1 receptor, which via NF-*κ*B activation ultimately leads to increased ABC transporter expression and activity levels at the blood-brain barrier. Created with BioRender.com.

TNF*α* is commonly involved in CNS inflammation (Probert et al., 1997; Fresegna et al., 2020; Raffaele et al., 2020). In 1992, Sharief and Thompson described increased TNF*α* levels in the cerebrospinal fluid from patients with multiple sclerosis that correlated with blood-brain barrier dysfunction (Sharief and Thompson, 1992). Maternal infections in guinea pigs led to TNF*α* release, which decreased Abcb1 function at the blood-brain barrier of the fetus, consequently rendering the fetal brain vulnerable to potentially teratogenic compounds (Iqbal et al., 2012, 2016). At later stages of development, TNF*α* signaling decreases Abcg2 activity at the rat blood-brain barrier (Harati et al., 2012). In adulthood, TNF*α* has differential effects on *Abcb1* and *Abcg2* expression levels and associated protein and activity levels, depending on exposure time and concentration. For example, we showed that acute short-term exposure of isolated rat brain capillaries to nanomolar concentrations of TNF*α* activated the TNF receptor 1, which activated endothelin converting enzyme (Fig. 7; Hartz et al., 2006). Endothelin converting enzyme activation, in turn, leads to the production of endothelin 1, which signals through the endothelin receptor B to activate the inducible nitric oxide synthase. NO stimulates protein kinase C*β*1 (PKC*β*1) and sphingosine release from the brain capillary membrane (Pilorget et al., 2007; Rigor et al., 2010). Sphingosine is phosphorylated by sphingosine kinase and binds to the sphingosine-1-phosphate receptor decreasing *Abcb1* and *Abcg2* mRNA levels and associated protein activity levels at the blood-brain barrier in vitro and in vivo (Hartz et al., 2004, 2006; Evseenko et al., 2007; Pilorget et al., 2007; von Wedel-Parlow et al., 2009; Hawkins et al., 2010; Heemskerk et al., 2010; Cannon et al., 2012; Harati et al., 2013). In addition, the PKC*β*1 activator 12-deoxyphorbol-13-phenylacetate-20-acetate significantly increases brain uptake of the ABCB1 substrate [3H]-verapamil in rats, indicating that downregulating *Abcb1* expression and protein activity enhances brain drug delivery (Rigor et al., 2010).

Long-term exposure (6 hours) of isolated rat brain capillaries to TNF*α* leads to endothelin 1 release, which in turn activates both endothelin receptor A receptors and endothelin receptor B receptors, stimulating NO release and activation of PKC*β*2. This signaling pathways results in downstream activation of NF-*κ*B, which translocates to the nucleus and induces transcription and translation of *Abcb1* at the blood-brain barrier (Rigor et al., 2010; Bauer et al., 2007; Mayati et al., 2017).

##### d. Prostaglandins

In 1995, Tishler et al. analyzed resected brain tissue from patients with medically intractable (refractory or drug-resistant) epilepsy and found increased *ABCB1* mRNA levels (Tishler et al., 1995). This led to the transporter hypothesis of refractory epilepsy, which states that ABCB1 overexpression at the blood-brain barrier in epilepsy restricts antiseizure brain drug uptake, thus, leading to antiseizure drug resistance (Tang et al., 2017). Much research has been done to understand the role of blood-brain barrier ABC transporters in epilepsy and lead to the partial unraveling of signaling pathways that control these transporters after seizures.

Release of glutamate in the CNS of patients with epilepsy is linked to seizure activity and subsequent CNS damage (Ronne-Engstrom et al., 1992). Extracellular glutamate upregulates *Abcb1* mRNA and associated protein levels in rat brain capillary endothelial cells in vitro and proposed that glutamate activates the N-methyl-D-aspartate receptor (NMDAR) and triggers a signaling cascade that increases Abcb1 expression at the blood-brain barrier (Zhu and Liu, 2004). At the same time, efflux of anticonvulsive drugs by ABC transporters at the blood-brain barrier was considered as one of the main causes of refractory epilepsy (van Vliet et al., 2005).

Since then, we and others have identified several signaling steps through which seizures upregulate ABC transporters at the blood-brain barrier. Specifically, seizure-induced glutamate release activates the NMDAR in brain capillaries (Hartz et al., 2019; Mohamed et al., 2019). NMDAR activation stimulates cytosolic phospholipase A2 to cleave arachidonic acid from triglycerides in the cell membrane (Hartz et al., 2019). Arachidonic acid is converted by cyclooxygenase 2 to prostaglandin H2, which is then converted to prostaglandin E2 by microsomal prostaglandin synthase 1, a process first described in isolated rat brain capillaries (Baba et al., 1985; Bauer et al., 2008b; Zibell et al., 2009; Schlichtiger et al., 2010; van Vliet et al., 2010; Soldner et al., 2019). Prostaglandin E2 activates the prostaglandin EP1 receptor in brain capillary endothelial cells and via NF-*κ*B activation ultimately leads to increased ABC transporter expression and activity levels (Pekcec et al., 2009; Soldner et al., 2019). Targeting signaling steps in this pathway has the potential to prevent *ABCB1* upregulation at the blood-brain barrier and thus overcome drug resistance in patients with epilepsy (Bauer et al., 2008b; Pekcec et al., 2009; Zibell et al., 2009; Schlichtiger et al., 2010; van Vliet et al., 2010; Hartz et al., 2019; Mohamed et al., 2019; Soldner et al., 2019). This pathway is active at the blood-brain barrier of patients with epilepsy and ALS (Avemary et al., 2013; Mohamed et al., 2019). In addition to epileptic seizures, morphine withdrawal also activates this pathway and upregulates *Abcb1* expression and associated protein activity levels at the rat blood-brain barrier (Yousif et al., 2012; Chaves et al., 2017). Other cell membrane lipids like ceramide 1-phosphate and other sphingolipids also stimulate this pathway and increase Abcb1 activity at the blood-brain barrier (Mesev et al., 2017). Taken together, prostaglandin signaling is a key pathway that regulates *ABCB1* and *ABCG2* and associated proteins at the blood-brain barrier.

Multiple drugs operate through modifying prostaglandin levels or activity. These include bimatoprost (glaucoma treatment), carboprost (induce uterine contractions), dinoprost (cervical dilation during labor), misoprostol (abortifacient, gastric ulcer treatment), and latanoprost (glaucoma treatment). The antibiotic cefmetazole can inhibit prostaglandin transport out of the brain across the blood-brain barrier (Akanuma et al., 2011). However, focused research on modifying prostaglandin activity to regulate blood-brain barrier activity is currently lacking.

##### e. Other cytokines

Several other cytokines are involved in transporter regulation at the blood-brain barrier but have not been studied extensively. For example, interleukin (IL) 1*β* decreases expression and activity levels of both *Abcb1* and *Abcg2* and associated proteins (Evseenko et al., 2007; Ronaldson et al., 2008; Robey et al., 2009; Ashraf et al., 2011). Another example includes members of the IL-6 family, including leukemia inhibitory factor and ciliary neurotrophic factor that stimulate NF-*κ*B signaling and increase Abcb1 activity at the blood-brain barrier (Monville et al., 2002; Evseenko et al., 2007; Ashraf et al., 2011). *ABCG2* expression levels, on the other hand, are decreased by IL-6 (Poller et al., 2010). While the underlying mechanisms of how cytokines affect transporters are not well understood, some data indicate that cytokine signaling alters caveolae in the brain capillary endothelium, which moves transporters from intracellular storage vesicles into the luminal membrane (Tome et al., 2016).

#### 2. Oxidative Stress

Oxidative stress occurs in many CNS disorders (Qosa et al., 2016a; Singh et al., 2019; Mendiola et al., 2020). Reactive oxygen species (ROS) generated during oxidative stress damage cells lead to cytokine release, which in turn affects cellular processes including transport (Mendiola et al., 2020). In 2002, Felix et al. first demonstrated that hypoxia-induced ROS increase Abcb1 mRNA and protein expression levels in rat brain capillary endothelial cells in vitro (Felix and Barrand, 2002; Neuhaus et al., 2014). Following this initial discovery, other groups also unraveled signaling pathways through which oxidative stress increases *Abcb1* expression and associated protein activity levels at the blood-brain barrier. For example, ROS stimulate the extracellular signal-regulated kinase (ERK) signaling cascade that includes protein kinase C, c-Jun, and Akt (Bauer et al., 2005; Miller et al., 2008). The key relay in this pathway is NF-*κ*B that, once activated, increases Abcb1 protein levels (Bauer et al., 2005; Miller et al., 2008; Qosa et al., 2016a; Grewal et al., 2017). In contrast, ROS-mediated ERK1 and ERK2 stimulation in mouse brain capillary endothelial cells in vitro induces *Abcg2* downregulation (Neuhaus et al., 2014; Grewal et al., 2017). ROS also oxidizes Kelch-like ECH-associated protein 1, which releases nuclear factor E2-related factor 2 (Nrf2) allowing its translocation into the nucleus. There, Nrf2 binds to the antioxidant response element in the promoter region of its target genes resulting in increased transcription. These target genes code for detoxification enzymes, antioxidant proteins, proteins involved in xenobiotic metabolism, and efflux transporters including Abcb1 (Klaassen and Slitt, 2005; Maher et al., 2005; Copple, 2010, 2012; Aleksunes and Klaassen, 2012). Nrf2 also activates p53 and stimulates the p38 mitogen-activated protein kinase (MAPK) cascade that activates NF-*κ*B, which increases *Abcb1* expression and associated protein levels in isolated rat brain capillaries (Wang et al., 2014; Grewal et al., 2017). On the other hand, oxidative stress activates pathways that decrease *ABCB1* expression and associated protein levels at the blood-brain barrier. For example, oxidative stress activates Abl and Src kinases that phosphorylate caveolin-1, which triggers internalization of both caveolin-1 and colocalized ABCB1 reducing *ABCB1* expression and protein activity levels (Hoshi et al., 2019).

If ROS affects transport, one might expect antioxidants like N-acetylcysteine to reverse this. Indeed, exposing cultured rat capillary endothelial cells or isolated rat brain capillaries to N-acetylcysteine reversed the ROS effects on the expression and activity of *Abcb1* and *Abcg2* and associated proteins (Zhu and Liu, 2004; Li et al., 2016; Zhou et al., 2019), which might provide a future clinical avenue.

#### 3. Clinical Relevance of Inflammatory and Oxidative Stress Signaling

Our research shows that, in epilepsy, seizure-induced glutamate release activates a prostaglandin-dependent signaling pathway leading to *Abcb1* and *Abcg2* upregulation at the blood-brain barrier (Bauer et al., 2008b; Pekcec et al., 2009; Zibell et al., 2009; Hartz et al., 2017). In addition, proinflammatory cytokines are increased in the blood and brain of patients with epilepsy indicating neuroinflammation. Cytokines that are upregulated in the brain of patients with epilepsy include IL1*α*, IL1*β*, IL6, and TNF*α*, all of which regulate blood-brain barrier ABCB1 and ABCG2 (Arend et al., 2018; de Vries et al., 2016; Gao et al., 2016; Mercado-Gomez et al., 2018; Kothur et al., 2019). Moreover, our and other data indicate that inflammation contributes to elevated protein levels and functional activity of ABCB1/Abcb1 and ABCG2/Abcg2 and is also involved in epileptogenesis (Hartz et al., 2004; Bauer et al., 2007, 2008a; Alyu and Dikmen, 2017; Rana and Musto, 2018). Neuroinflammation exacerbates seizures and increased expression levels of drug efflux transporters in the brain endothelium could hinder antiseizure drugs from entering the brain (Cox et al., 2001; van Vliet et al., 2006). Uncontrolled seizures in patients cause more neuroinflammation driving a vicious cycle of disease progression and drug resistance. In addition to epilepsy, neuroinflammation is part of many other CNS disorders. In glioblastoma, for example, tumor cells stimulate microglia to release proinflammatory cytokines such as IL1*β*, IL6, TNF*α*, and prostaglandins (Maruno et al., 1997; Matsuo et al., 2001; Samaras et al., 2007; Schwartzbaum et al., 2007, 2017; Wang et al., 2009; Jiang et al., 2017; Couto et al., 2019; Gao et al., 2019; Ham et al., 2019). In this context, microglia help form a proinflammatory tumor microenvironment that is conducive to gliomagenesis and tumor growth and progression including tumor migration and invasion (Maruno et al., 1997; Matsuo et al., 2001; Samaras et al., 2007; Wang et al., 2009; Desmarais et al., 2015; Lepore et al., 2018). In addition, cytokines transcriptionally upregulate *ABCB1*/*ABCG2* and enhance translation of their associated proteins at the blood-brain barrier, which restricts anticancer drug delivery into the brain. Blocking cytokine signaling prevented tumor growth and invasion, which improved survival in glioblastoma animal models (Desmarais et al., 2015; Kast et al., 2017; Lamano et al., 2019). Thus, such treatment approaches could help prevent or reverse *ABCB1*/*ABCG2* overexpression and potentially improve drug delivery and efficacy in patients.

Brain levels of proinflammatory cytokines are also increased in neurodegenerative diseases, including Alzheimer’s disease (Akiyama et al., 2000; Cai et al., 2014). Amyloid *β* (A*β*), a neurotoxic peptide and one hallmark of Alzheimer’s disease, activates microglia, which in turn generate and release IL1, IL6, TNF*α*, and prostaglandins into the brain parenchyma. These inflammatory mediators, in turn, activate NF-*κ*B signaling, which leads to more inflammation and even higher A*β* levels in Alzheimer’s disease patients, ultimately leading to neuronal death (Bauer et al., 1991; Buxbaum et al., 1992; Strauss et al., 1992; Mackenzie et al., 1995; Pasinetti and Aisen, 1998; Ringheim et al., 1998; Ho et al., 1999; Kitamura et al., 1999; Meda et al., 1999; Tarkowski et al., 1999; Yates et al., 2000; Combs et al., 2001; Haas et al., 2002; McGeer and McGeer, 2003; Sochocka et al., 2013; Cai et al., 2014; Mosher and Wyss-Coray, 2014; Heppner et al., 2015; Bhattacharya et al., 2020).

While levels of proinflammatory mediators are increased, *ABCB1*/*Abcb1* and *ABCG2*/*Abcg2* expression and associated protein activity levels are decreased in animal models of Alzheimer’s disease as well as in patients with Alzheimer’s disease (Vogelgesang et al., 2002, 2004; Hartz et al., 2010c, 2012, 2018; Wijesuriya et al., 2010; Jeynes and Provias, 2011b; van Assema et al., 2012b; Mehta et al., 2013; Carrano et al., 2014; Chiu et al., 2015; Wang et al., 2016; Bauer et al., 2017; Kannan et al., 2017; Shubbar and Penny, 2018; Al-Majdoub et al., 2019). At this point it is unclear if neuroinflammation contributes to the loss of ABCB1/ABCG2 proteins at the blood-brain barrier in Alzheimer’s disease.

Research and development of anti-inflammatory strategies for neurologic disorders that involve the blood-brain barrier are currently in progress. Currently, 14 clinical trials are reported by clinicaltrials.gov as recruiting, enrolling, completed, or in planning. However, none of these trials address blood-brain barrier transporters.

### C. Receptor Tyrosine Kinases and Growth Factor Signaling

Growth factors, cytokines, and hormones activate receptor tyrosine kinases (RTK) that are critical in survival and apoptosis (Robinson et al., 2000). RTKs have a hydrophobic transmembrane domain that connects the extracellular N terminus with the ligand binding domain and the intracellular C terminus containing the catalytic kinase domain (Hubbard, 1999; Zwick et al., 2001). Ligands, like growth factors, cytokines, and hormones, bind to the ligand binding domain and induce receptor dimerization and rapid activation of the kinase domain. Autophosphorylation of the receptor allows signal transfer through the cell membrane. The phosphorylated receptor interacts with adaptor proteins that act as linkers to downstream kinases, such as Src or phospholipase C. These kinases further activate a network of redundant pathways with feedback loops, crosstalk, and compensatory mechanisms (Zwick et al., 2001; Lemmon and Schlessinger, 2010). Mutations in RTKs or downstream signaling partners are implicated in the development and progression of cancer (Zwick et al., 2001). RTK signaling cascades regulate proliferation and modify protein expression and activity (Lemmon and Schlessinger, 2010). Several growth factors and their respective RTKs regulate ABCB1 and ABCG2 at the blood-brain barrier, including epidermal growth factor (EGF), platelet-derived growth factor (Smits et al., 1989; Bleau et al., 2009b), transforming growth factor beta (Dohgu et al., 2004; Baello et al., 2014), and vascular endothelial growth factor (VEGF). In general, activation of RTKs and downstream signaling cascades increases expression levels of blood-brain barrier ABCB1 and ABCG2 but also of other transporters such as Oatp1a1 (Ronaldson et al., 2011). In contrast, inhibition of this signaling decreases transporter expression levels.

The FDA has approved several RTK inhibitors for anticancer use, including axitinib, cabozantinib, lenvatinib, nintedanib, and others (Hou et al., 2021). Effects of such drugs on the blood-brain barrier have not been characterized, and RTK agonists are even less investigated.

#### 1. Janus Kinase and Signal Transducer and Activator of Transcription 3 Cascade

**Fig. 8:**
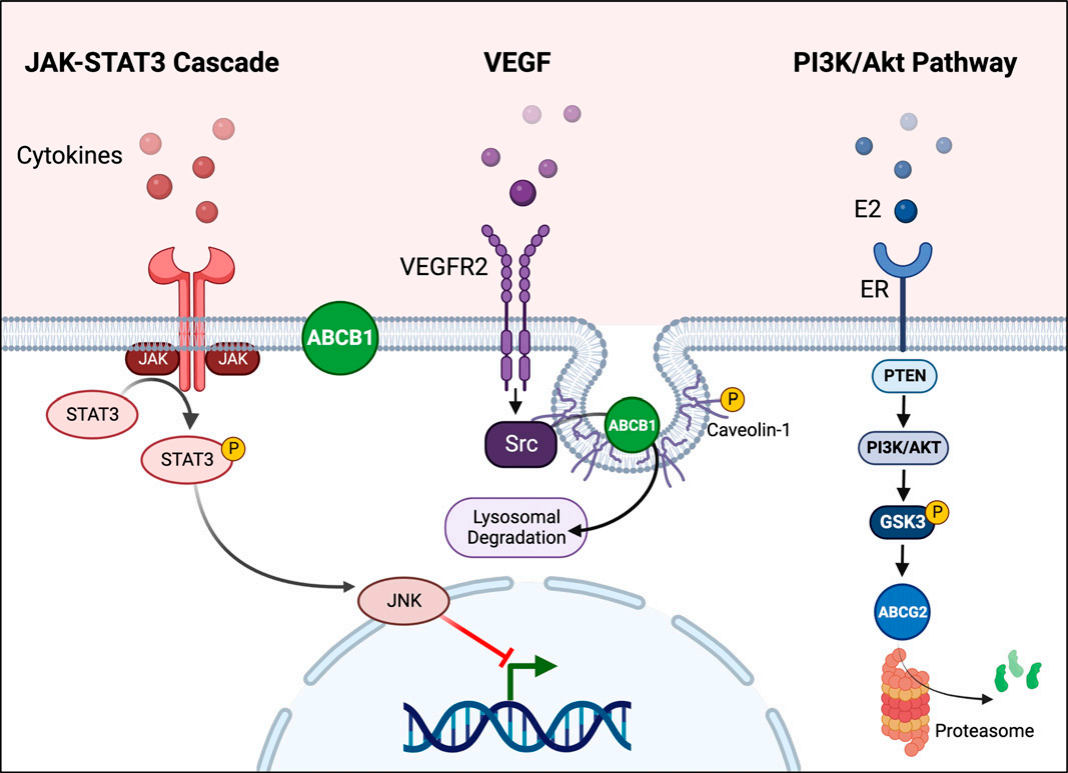
Regulation of ABCB1 by JAK-STAT3. Cytokines activate the JAK-STAT3 cascade, which leads to phosphorylation of STAT3, which leads to activation of the c-Jun NH2 terminal kinase that in turn deactivates c-Jun and reduces *Abcb1* mRNA expression levels. Regulation by VEGF. VEGF signals through VEGFR2 to activate the nonreceptor tyrosine kinase Src. Activation of Src then induces phosphorylation of caveolin-1, which is followed by Abcb1 internalization and lysosomal degradation of the transporter. Regulation by the PI3K/Akt pathway. E2 signaling through ER*β* inhibits the PTEN/PI3K/Akt/GSK3 pathway, which in turn leads to proteasomal degradation of Abcg2. Created with BioRender.com.

The Janus kinase and signal transducer and activator of transcription 3 (JAK-STAT3) cascade is commonly activated by cytokines (Fig. 8). Downregulation and inhibition of JAK1, STAT3, or phosphorylated STAT3 downregulate ABCB1 at the blood-brain barrier (Jagadeeshan et al., 2017). Similar effects occur after p38 MAPK inhibition. Specifically, inhibiting p38 MAPK in epileptic rats decreased *Abcb1* expression and associated protein activity levels, which correlated with increased brain drug uptake (Shao et al., 2016). The signaling molecules JNK, ERK, and c-Jun are also involved in this cascade, and activation of the c-Jun NH2 terminal kinase deactivates c-Jun and reduces *Abcb1* mRNA expression levels (Zhou et al., 2006; Ronaldson et al., 2008). However, activating ERK and c-Jun with EGF via EGFR induces Abcb1 and Abcg2 expression at the blood-brain barrier (Bauer et al., 2005; Evseenko et al., 2007; Nakanishi and Ross, 2012; Munoz et al., 2014).

Currently approved JAK inhibitors include tofacitinib and ruxolitinib that are used for the treatment of rheumatoid arthritis and autoimmune diseases (Taylor, 2019). Other indications include ulcerative colitis, myelofibrosis, and atopic dermatitis (Hu et al., 2021). However, targeting the JAK-STAT3 signaling cascade for blood-brain barrier manipulation is little explored.

#### 2. Vascular Endothelial Growth Factor

VEGF activates its receptors VEGFR2 and Flk-1 that in turn activate the nonreceptor tyrosine kinase Src (Fig. 8; Agarwal et al., 2011). Src activation induces phosphorylation of caveolin-1 followed by Abcb1 internalization and degradation (Hawkins et al., 2010; Miller and Cannon, 2014; Qosa et al., 2015). Phosphorylated caveolin-1 colocalizes with Abcb1 in the plasma membrane and initiates Abcb1 endocytosis and vesicular trafficking to the endosome or lysosome. There, Abcb1 is either recycled and trafficked back to the plasma membrane or, as in most cases, undergoes lysosomal degradation (Tome et al., 2015).

EGFR is highly expressed in some cancers, and EGFR inhibitors are used to block cancer cell growth. In glioblastoma patients, currently available EGFR inhibitors do not provide a survival benefit but could be used to overcome blood-brain barrier ABCB1/ABCG2-mediated drug resistance via transporter internalization/degradation (de Gooijer et al., 2018b; Kim et al., 2019b). In particular, EGFR inhibitors with improved brain partitioning, like buparlisib, might be useful to increase drug delivery into the brain and thus be beneficial in the treatment of glioblastoma patients (de Gooijer et al., 2018b).

#### 3. PI3K/Akt/mTOR Pathway

In cancer, including gliomas, the PI3K/Akt/mTOR signaling pathway is dysregulated due to the loss of the tumor suppressor PTEN or constitutively active PI3K and Akt mutants. PTEN loss and PI3K/Akt mutations induce proliferation and prevent apoptosis of cancer cells and are associated with migration, invasion, and resistance to both radiation and chemotherapy (Balsara et al., 2004; Chen et al., 2005; Mellinghoff et al., 2005; Tang et al., 2006; Jiang et al., 2007; Cancer Genome Atlas Research Network, 2008; Verhaak et al., 2010; Song et al., 2012; Zhang et al., 2015; Wang et al., 2017). PI3K/Akt/mTOR also regulates transporters and in several cancers PI3K/Akt/mTOR signaling leads to transporter-mediated drug resistance. For example, constitutive overactivity of the PI3K/Akt pathway induces *ABCB1* and *ABCG2* expression and their associated proteins’ translocation to the plasma membrane of brain microvasculature endothelial cells, which increases drug resistance of brain tumors (Takada et al., 2005; Bleau et al., 2009b; Agarwal et al., 2011; Huang et al., 2013; Huang et al., 2014b). In this regard, we showed that estradiol activation of ER*β* activates PTEN, leading to PI3K/Akt inactivation followed by GSK3 phosphorylation. Phosphorylated GSK3 then induces ABCG2 internalization and proteasomal degradation (Fig. 8; Hartz et al., 2010a,b). Similarly, PI3K inhibition with LY294002 stimulates ABCG2 transporter internalization and degradation (Mogi et al., 2003; Takada et al., 2005; Bleau et al., 2009a).

Until recently, the cause of transporter upregulation in cancer was unknown. Matsumoto et al. (1991) found *ABCB1* overexpression in glioblastoma samples from patients after initial treatment and concluded that glioblastoma acquires ABCB1-mediated resistance during treatment (Matsumoto et al., 1991). However, transporter overexpression could also be due to pathway dysregulation. In support of this model, RTK signaling components, including downstream kinases like PI3K, are mutated in 95% of glioblastoma patients (Schlessinger, 2000; Brennan et al., 2013; Eskilsson et al., 2018). Overactive PI3K signaling drives tumor progression and upregulates *ABCB1* and *ABCG2* expression and associated protein activity in tumors and at the blood-brain barrier, contributing to drug resistance (Schlessinger, 2000; Shinojima et al., 2003; Brennan et al., 2013; Eskilsson et al., 2018). Specifically, activation of the PI3K/Akt signaling cascade through increased phosphorylation of PI3K and Akt, PI3K/Akt overexpression or loss of PTEN, significantly increases *ABCB1* and *ABCG2* expression and associated protein activity at the blood-brain barrier (Brennan et al., 2013; Huang et al., 2013, 2014a). Thus, inhibiting PI3K, mTOR, or upstream RTKs holds the potential to attenuate transporter upregulation (Huang et al., 2013; de Gooijer et al., 2018c).

In tumor cells and at the blood-brain barrier, ABCB1 and ABCG2 restrict the uptake of anticancer drugs, and transporter expression levels were thought to correlate with the extent of multidrug resistance (Marchetti et al., 2008; Agarwal et al., 2010, 2011; de Vries et al., 2012; Laramy et al., 2017; de Gooijer et al., 2018c). This transporter-centric view on multidrug resistance in cancer, however, is controversial.

### D. Other Pathways

Several other pathways that regulate blood-brain barrier ABCB1/ABCG2 have been identified in recent years and are summarized in the following sections.

#### 1. Adenosine

**Fig. 9:**
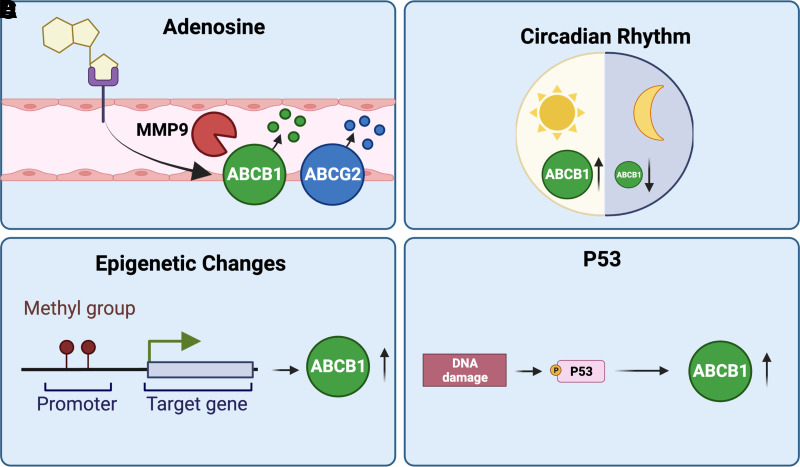
Other signaling pathways. Diagram showing several other signaling pathways identified to regulate ABCB1 and/or ABCG2 at the blood-brain barrier: (A) adenosine, (B) circadian rhythm, (C) epigenic changes, and (D) P53. Created with BioRender.com.

Adenosine receptor A_2_ and adenylate cyclase, both key components of the adenosine signaling pathway, exist at the blood-brain barrier (Fig. 9A; Kalaria and Harik, 1986). In 2016, Kim and Bynoe (2016) demonstrated that activation of the adenosine receptor A_2A_ in hCMEC/D3 cells in vitro decreases *ABCB1* and *ABCG2* expression and associated protein activity through membrane metalloprotease 9-mediated cleavage followed by ubiquitination and proteasomal degradation (Kim and Bynoe, 2016). A_2A_ activation decreases the expression of tight junction proteins, which increases paracellular permeability (Kim and Bynoe, 2015). The adenosine receptor A_2B_ has a low affinity for adenosine and to be activated requires high adenosine concentrations that are usually associated with pathologic conditions, such as brain tumors (Fredholm et al., 2001; Hasko et al., 2009; Aherne et al., 2011). Jackson et al. (2016, 2018) demonstrated that A_2B_ inhibition disrupts blood-brain barrier function and improves brain drug uptake in rats (Jackson et al., 2016, 2018). Later studies showed that A_2B_ signaling through protein kinase A and phospholipase C increases ABCB1 protein levels in endothelial cells, thereby contributing to drug resistance (Hasko et al., 2009; Aherne et al., 2011; Yan et al., 2019a).

Adenosine levels are increased in various pathologic conditions including brain cancer (Hasko et al., 2009; Aherne et al., 2011; Jackson et al., 2016; Yan et al., 2019a). Several preclinical studies suggest that increased adenosine levels could drive tumor invasion and drug resistance in glioblastoma models (Yan et al., 2019a,b; Zavala-Tecuapetla et al., 2020). Preclinical studies also suggest that adenosine inhibition improves drug brain uptake and efficacy in animal glioblastoma and epilepsy models. In a 2017 pilot study with patients with cardiovascular disease, A_2A_ inhibition increased brain levels of the imaging reagent (99m)Tc-sestamibi (Jackson et al., 2017). However, A_2A_ inhibition did not translate into increased temozolomide levels in a phase 1 clinical trial in glioblastoma patients (Jackson et al., 2018). At this point the therapeutic value of adenosine receptor inhibition to regulate blood-brain barrier transporters with the goal of improving brain drug levels needs further evaluation.

#### 2. Circadian Rhythm

The circadian rhythm regulates multiple physiologic processes, including metabolism and transport, which affects drug delivery and elimination and,f thus, efficacy (Fig. 9B; Erol et al., 2001; Okyar et al., 2012; Filipski et al., 2014; Kervezee et al., 2014; Savolainen et al., 2016). At the molecular level, the circadian rhythm is controlled by a complex system of transcription factors including the main circadian regulator CLOCK, its heterodimer BMAL1, PAR domain basic leucine zipper proteins, Period, and E4BP4, a transcriptional activator protein that follows an opposing oscillating cycle and acts as a negative regulator during the sleep phase (Gekakis et al., 1998; Murakami et al., 2008). These proteins regulate a number of processes including the circadian oscillations of metabolizing enzymes and drug transporters.

In this regard, Zhang et al. (2018) first reported that xenobiotic efflux at the blood-brain barrier in fruit flies (*Drosophila melanogaster*) underlies circadian rhythm regulation. Specifically, they studied Mdr65, which is a pesticide-resistance protein with homology to ABCB1, along with Mdr49. Mdr65 activity is regulated through opposing variation of intracellular Mg^2+^ and Ca^2+^ concentrations without changing transporter expression. Importantly, increased Mdr65-mediated efflux during the active period of the fruit fly decreased brain accumulation of xenobiotics (Zhang et al., 2018). The oscillating changes in efflux are repressed in Period knockout flies, further supporting circadian rhythm regulation of efflux transporter activity. Furthermore, Abcb1 activity at the blood-brain barrier of mice and ABCB1 activity in hCMEMC/D3 cells in vitro also underly circadian rhythm regulation (Zhang et al., 2021). Of particular note is that circadian oscillation of ABCB1 intestinal expression in primates (*Macaca fascicularis*) altered the pharmacokinetics of its substrates, suggesting a similar principle operating in the brain of higher mammal (Iwasaki et al., 2015).

In this regard, in the 1970s researchers discovered that mice with leukemia responded better to treatment when anticancer drug therapy was adjusted to the circadian rhythm (Haus et al., 1972). Thus, a dosing schedule following the circadian rhythm has the potential to improve brain uptake and efficacy of CNS therapeutics. Several studies show that brain uptake of the Abcb1 substrate quinidine is increased during the resting phase of mice compared with the active phase, when Abcb1 activity is increased (Kervezee et al., 2014; Savolainen et al., 2016). Human patients had higher serum drug levels and better seizure control when phenytoin was dosed at night compared with the morning (Yegnanarayan et al., 2006). These findings suggest that decreased ABCB1 activity during the resting phase—daytime in rodents, nighttime in patients—is sufficient to improve drug brain uptake and efficacy while decreasing side effects (Yegnanarayan et al., 2006; Filipski et al., 2014).

#### 3. Epigenetic Markers

Epigenetic markers include methylation and hydroxymethylation of target gene DNA and methylation, acetylation, phosphorylation, ubiquitylation, and sumoylation of associated histones that alter gene transcriptional activity without changing DNA sequence (Fig. 9C; Liberman et al., 2019). Regarding *ABCB1*, promotor (de)methylation is an important regulator of transporter expression (Scotto, 2003; Sarkadi et al., 2006; Nakanishi and Ross, 2012). *ABCB1* promotor methylation increases the association of chromatin with deacetylated histones and methyl-CpG-binding protein 2, which represses transcription and, in turn, reduces *ABCB1* mRNA and protein expression levels (El-Osta et al., 2002). Promotor demethylation triggers the release of methyl-CpG-binding protein 2 and causes chromatin relaxation, which enables *ABCB1* gene transcription (El-Osta et al., 2002). While histone acetylation does not activate the *ABCB1* promotor, it induces *ABCB1* transcription and causes transporter overexpression (El-Osta et al., 2002; Nakanishi and Ross, 2012). Several groups have investigated histone acetyltransferase and histone deacetylase (HDAC) and their effects on *ABCB1* expression. HDAC inhibition specifically increases acetylation of histone H4, which regulates the expression of Rab GTPases that stimulate vesicular trafficking of ABCB1 protein to the membrane (Noack et al., 2016). HDAC inhibition and histone acetyltransferase activation combined activate the *ABCB1* promotor (Jin and Scotto, 1998; Sarkadi et al., 2006; You et al., 2019). You et al. (2019) showed that HDAC inhibitors increase *ABCB1* mRNA and protein levels in cultured hCMEC/D3 cells, accompanied by increased AhR binding to the *ABCB1* promotor, indicating a coregulatory mechanism.

Another epigenetic pathway involves melatonin, a hormone that controls the sleep-wake cycle and methylates the *ABCG2* promotor, which ultimately leads to decreased ABCG2 protein levels in brain tumor stem cells. Since melatonin is a DNA methyltransferase substrate, methyltransferase inhibitors prevent *ABCG2* downregulation (Martin et al., 2013). Recently, Jumnongprakhon et al. (2017) identified an alternative melatonin signaling pathway that regulates *Abcb1* in primary rat brain microvascular endothelial cells. They showed that melatonin reverses methamphetamine-induced reduction in *Abcb1* mRNA expression and associated protein levels at the blood-brain barrier by preventing internalization, ubiquitination, and proteasomal degradation (Jumnongprakhon et al., 2017). Since melatonin is not an Abcb1 substrate, competitive inhibition is unlikely to contribute to these effects (Tran et al., 2009).

Gene amplification, alternative promotors, and multiple transcription start sites are important *ABCB1* regulators in the context of bacterial antibiotic resistance and cancer multidrug resistance; their role at the blood-brain barrier is unknown (Nakanishi and Ross, 2012).

Epigenetic changes are common in cancer cells, where they drive tumor progression and drug resistance (Hegi et al., 2005; Dong and Cui, 2019). In this regard, anticancer drugs, specifically DNA alkylating agents, increase DNA methylation, which causes double-strand breaks and leads to apoptosis. While this is the main mechanism of action of DNA alkylating agents, drugs from this category also change expression levels of other genes, including *ABCB1* and *ABCG2* (Munoz et al., 2015). This might explain why temozolomide and carmustine, two clinically used alkylating agents for the treatment of glioblastoma, decrease their own brain distribution and efficacy in glioblastoma patients (Valtonen et al., 1997; Stupp et al., 2005). However, opposing effects of alkylating agents have been described in glioblastoma cells (Riganti et al., 2013).

#### 4. p53

p53 (gene: *TP53*) is one of the best-studied tumor suppressor proteins. Mutations and loss of p53 in different cancer types upregulate transcription and translation of *ABCB1* (Fig. 9D; Bush and Li, 2002; Marroni et al., 2003; Sarkadi et al., 2006). Wild-type p53 decreases Ras/Raf signaling and phospholipase C activity, which increases ABCB1 expression levels (Chin et al., 1992; Bush and Li, 2002; Scotto, 2003). At the blood-brain barrier, DNA damage induces ataxia-telangiectasia-mutated kinase, which, in turn, activates p53, p38, and NF-κB, leading to ABCB1 overexpression (Bush and Li, 2002; Bleau et al., 2009b; Miller, 2015). Interestingly, the *TP53* promoter is activated by intracellular Aβ, an ABCB1 substrate (Ohyagi et al., 2005).

*TP53*, which encodes p53, is the most mutated gene in cancer, including 30% of all brain tumors (Cancer Genome Atlas Research Network, 2008; Brennan et al., 2013; Duffy et al., 2017). TP53 mutations result in p53 loss-of-function, which dysregulates cell cycle progression, proliferation, and tumor growth.

## IV. Overall Clinical Implication

**Fig. 10:**
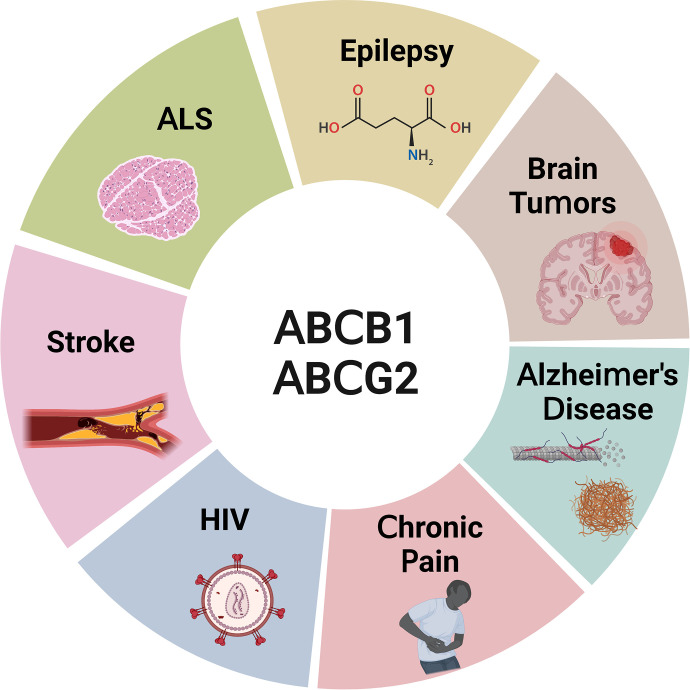
Clinical implications. Overview of diseases where ABCB1 and/or ABCG2 are changed and affect the progression and treatment of the respective disease. Created with BioRender.com.

Approximately 1 billion people worldwide and 100 million people in the United States suffer from CNS diseases, accounting for 6.3% of total global disease burden [World Health Organization (WHO), 2006; Gooch et al., 2017]. Many CNS diseases are difficult to treat, and the economic and social impact of treatment failure is significant (WHO, 2006; Gooch et al., 2017). Consequently, new therapeutic strategies are urgently needed. Two main obstacles exist to successful treatment of CNS disorders. First, the efflux transporters ABCB1 and ABCG2 at the blood-brain barrier prevent access of drugs to the brain and thus significantly interfere with the treatment of CNS diseases (Schinkel et al., 1994; Kim et al., 1998b; de Vries et al., 2007). Second, changes in the blood-brain barrier contribute to disease pathology that further restricts drug uptake into the brain, adding another layer of intricacy to the successful treatment of CNS diseases. Next we discuss some of the distinct changes that occur at the blood-brain barrier in patients with CNS disease or in animal disease models and highlight how those changes affect the progression and treatment of the respective disease (Fig. 10).

### A. Epilepsy

The WHO estimates that about 46 million people worldwide suffer from epilepsy, a disease characterized by recurring, unprovoked seizures that also impair cognition and sleep (Beghi, 2020). Approximately one-third of epilepsy patients do not respond to pharmacological treatment with antiseizure drugs and thus suffer from refractory, drug-resistant epilepsy and uncontrolled seizures (Kwan and Brodie, 2000). One major contributor to this drug resistance is ABCB1 and potentially other drug efflux transporters including ABCG2 at the blood-brain barrier, which prevent the delivery of antiseizure drugs into the brain (Cox et al., 2001; van Vliet et al., 2006; Tang et al., 2017). The significance ABCB1 plays in refractory epilepsy is further amplified by the fact that ABCB1 is upregulated at the blood-brain barrier of patients with refractory epilepsy (Tishler et al., 1995; Dombrowski et al., 2001). *Abcb1* is overexpressed specifically in epileptogenic brain regions of chronic epileptic rats (van Vliet et al., 2006). In a case report, Iannetti et al. demonstrated that the ABCB1 inhibitor verapamil increased the efficacy of antiseizure drugs in a boy with epilepsy (Iannetti et al., 2005). However, the use of ABCB1 inhibitors is currently not a viable clinical strategy due to severe adverse effects.

### B. Brain Tumors

Brain tumors account for 2% of all cancer cases worldwide but disproportionately contribute to cancer morbidity and mortality (Kleihues et al., 2014). In the United States, approximately 77,000 patients are newly diagnosed with a brain tumor every year, which makes brain tumors the most common and deadliest cancer in children and the sixth most common cancer in adults (Ostrom et al., 2019). Treatment options are limited and mostly involve surgical resection and radiotherapy (Stupp et al., 2005). Chemotherapy of primary and secondary brain tumors is often unsuccessful, in part because ABCB1 and ABCG2 at the blood-brain barrier restrict access to a wide range of anticancer drugs to the brain and impair their efficacy (de Vries et al., 2012; Taskar et al., 2012; de Gooijer et al., 2018d; Sorf et al., 2018). The situation is especially dire for patients with glioblastoma multiforme, the most common malignant primary brain tumor (Ostrom et al., 2018). Median survival after diagnosis is 15 to 23 months, and fewer than 7% of glioblastoma patients survive 5 years or longer (Ostrom et al., 2018, 2019). While many anticancer drugs are promising in vitro, their efficacy in vivo and in clinical trials has been marginal at best, due to ABCB1- and ABCG2-mediated efflux at the blood-brain barrier. Recently, Kim et al. demonstrated and visualized this “ABC challenge” in an elegant study (Kim et al., 2018, 2019a). In brief, Kim and coworkers showed that the MDM2 inhibitor SAR405838 significantly decreased the viability of patient-derived glioblastoma cell lines (Kim et al., 2018). In addition, SAR405838 prevented growth of glioblastoma cells injected into the flanks of immunocompromised mice (Kim et al., 2018). In contrast, when the tumor cells were injected into the brain, the drug did not reach the brain and, therefore, had no beneficial therapeutic effect on survival in the orthotopic xenograft model (Kim et al., 2018). The data further showed that SAR405838 is both an ABCB1 and ABCG2 substrate (Kim et al., 2019a). Even though SAR405838 was effective in vitro and in flank models, ABCB1- and ABCG2-mediated efflux at the blood-brain barrier prevented drug entry into the brain, rendering the compound ineffective (Kim et al., 2019a).

Diffuse intrinsic pontine glioma (DIPG) is another example of a CNS tumor. DIPG is the most common brain tumor in children (Ostrom et al., 2019). DIPGs reside in the brain stem and, therefore, cannot be surgically removed or biopsied (Ostrom et al., 2019). Consequently, to date, little information is available on the molecular composition of DIPGs, hindering the development of targeted therapies. Data from recent studies with animal DIPG models indicate that blood-brain barrier ABC transporters contribute to the low efficacy of chemotherapy (Veringa et al., 2013; Becher and Wechsler-Reya, 2014; Chung et al., 2014; Duchatel et al., 2019; Chaves et al., 2020). Similar data have also been obtained for secondary, metastatic brain tumors of lung and breast cancer as well as melanoma. Several research groups have also shown increased uptake of fluorescent dextrans into brain metastases exists, suggesting a disrupted blood-brain/tumor barrier (Palmieri et al., 2009; Lockman et al., 2010; Taskar et al., 2012; Mittapalli et al., 2016; Osswald et al., 2016; Terrell-Hall et al., 2017; Mohammad et al., 2018). However, de Gooijer et al. (2021) and other groups have demonstrated that “ATP-binding cassette transporters restrict drug delivery and efficacy against brain tumors even when blood-brain barrier integrity is lost,” suggesting that ABCB1 and ABCG2 overcome barrier leakage (Thomas et al., 2009; Adkins et al., 2013; Dudek et al., 2013; Li et al., 2013; Ballard et al., 2016; Vaidhyanathan et al., 2016; Yang et al., 2016; Gampa et al., 2018, 2019; Ippen et al., 2019).

In addition to the blood-brain barrier, ABCB1 is also upregulated in tumor tissue samples from brain tumor patients. One underlying mechanism for ABCB1 upregulation in cancer cells is the amplification of the *ABCB1*-containing 7q21 chromosomal region, which confers multidrug resistance (Genovese et al., 2017). Thus, *ABCB1* copy number plays an important role in cancer-associated drug resistance. In glioblastoma, ABCB1 protein expression levels, measured with Western blotting and immunohistochemistry, were increased in patient samples from the second resection compared with those from the first resection (Matsumoto et al., 1991). ABCB1 expression increased in astrocyte-derived glioma cells compared with healthy human astrocytes (Spiegl-Kreinecker et al., 2002; Marroni et al., 2003). The increase in ABCB1 expression in glioblastoma patient samples may be mainly due to increased expression at the blood-tumor barrier, and transporter expression levels in tumor cells seem to play a minor role in drug resistance (Tanaka et al., 1994; Tews et al., 2000; Veringa et al., 2013).

### C. Alzheimer’s Disease

Alzheimer’s disease is the leading cause of dementia worldwide. In the USA, an estimated 6.5 million people over the age of 65 suffer from the disease (Alzheimer's Association, 2022). One hallmark of Alzheimer’s disease is the accumulation and aggregation of A*β* in the brain (Murphy and LeVine, 2010; Serrano-Pozo et al., 2011). The mechanism underlying brain A*β* accumulation is not fully understood, but data from recent studies suggest that loss of ABC transporters at the blood-brain barrier impairs A*β* clearance from the brain, contributing to an imbalance between A*β* production and clearance.

In 2001, Lam et al. (2001) were the first to show that ABCB1 transports A*β* in vitro (Lam et al., 2001; Kuhnke et al., 2007; Hartz et al., 2010c; Callaghan et al., 2020). Abcb1 cooperates with low-density lipoprotein receptor-related protein-1 (LRP1) located in the abluminal membrane of the brain capillary endothelium where it shuttles A*β* from the brain into brain capillary endothelial cells (Shibata et al., 2000; Deane et al., 2004; Storck et al., 2018). Additional data indicate that ABCB1 then clears A*β* from the endothelial cell into the blood (Cirrito et al., 2005; Hartz et al., 2010c; Mawuenyega et al., 2010; Hartz et al., 2018; Krohn et al., 2018).

In 2002, Vogelgesang and colleagues (2002) showed in brain tissue samples from nondemented elderly patients (n = 243; 50–91 years) that blood-brain barrier *ABCB1* expression and associated protein levels are decreased in areas with high A*β* load (Vogelgesang et al., 2002). Such a reduction in transporter levels also occurs in animal models of Alzheimer’s disease and in Alzheimer’s disease patients (Vogelgesang et al., 2004; Wijesuriya et al., 2010; Jeynes and Provias, 2011a; van Assema et al., 2012a; Mehta et al., 2013; Carrano et al., 2014; Chiu et al., 2015; Wang et al., 2016; Bauer et al., 2017; Kannan et al., 2017; Shubbar and Penny, 2018; Al-Majdoub et al., 2019). ABCB1 loss is particularly pronounced in areas directly surrounding A*β* plaques (Jeynes and Provias, 2011a; Chiu et al., 2015). Our group has shown that exposing isolated capillaries from mice and rats to nanomolar concentrations of human A*β* 40 decreases Abcb1 protein expression and activity levels (Hartz et al., 2010c, 2016). This effect is abolished by inhibiting ubiquitination, transporter internalization, and proteasomal degradation (Akkaya et al., 2015; Hartz et al., 2016, 2018; Ding et al., 2021). In addition to posttranslational modifications, other pathways including RAGE and NF-*κ*B also regulate *Abcb1* expression and associated protein levels at the blood-brain barrier in response to A*β* exposure (Park et al., 2014). While the role ABCB1 plays in A*β* clearance from the brain is well established, potential involvement of ABCG2 is less clear. In brain slices from Alzheimer’s disease patients capillaries surrounding A*β* plaques have decreased ABCG2 protein levels compared with cognitive normal controls (Carrano et al., 2014). However, other groups reported increased ABCG2 protein levels at the blood-brain barrier of Alzheimer’s disease patients (Xiong et al., 2009) or found no changes at all (Wijesuriya et al., 2010). Thus, currently it is unclear if ABCG2 is involved in A*β* clearance from the brain (Pahnke et al., 2008; Hartz et al., 2010c; Krohn et al., 2011). The involvement of other ABC transporters such as ABCA1 or ABCC1 in Alzheimer’s disease has been reviewed in detail by Wolf et al. (2012).

### D. Chronic Pain

Chronic pain is one of the leading causes of disability and significantly impairs patients’ ability to participate in daily activities (Gooch et al., 2017; Vlaeyen et al., 2018). Opioids (natural and synthetic) are commonly used to treat chronic pain and are also, coincidentally, ABCB1 substrates (Letrent et al., 1999; Dagenais et al., 2004; Bauer et al., 2006; Sharma and Ali, 2006; Hassan et al., 2007; Yousif et al., 2008, 2012; Chaves et al., 2016; Schaefer et al., 2018). For example, oxycodone, morphine, and methadone are weak ABCB1 substrates and therefore cross the blood-brain barrier and exert activity on the CNS (Gibbs et al., 2018). Some centrally active opioids such as oxycodone, morphine, or fentanyl, induce Abcb1 expression, diminishing their own brain uptake and efficacy (Letrent et al., 1999; Dagenais et al., 2004; Sharma and Ali, 2006; Hassan et al., 2007; Yousif et al., 2008, 2012; Chaves et al., 2016; Schaefer et al., 2018). Other opioids such as loperamide and naldemedine are more active Abcb1 substrates and thus only have low brain uptake, which minimizes their central action and side effects (Watari et al., 2019). Loperamide has a four times higher Abcb1-mediated transport rate than methadone, which significantly restricts its brain uptake and makes loperamide a safe and effective drug for diarrhea treatment without CNS side effects (Gibbs et al., 2018). Of potential note is that *λ*-carrageenan-induced inflammatory pain increased brain uptake and antinociception of codeine (Hau et al., 2004). This suggests that inflammatory pain may be an important consideration in therapeutic drug dosing, potential adverse effects, and neurotoxicity.

### E. Human Immunodeficiency Virus

Major breakthroughs in human immunodeficiency virus (HIV) treatment have transformed this originally deadly disease into a manageable chronic condition. The development of several classes of antiretroviral drugs with good safety and efficacy profiles significantly improved and prolonged the lives of HIV patients (Kwon et al., 2020). However, one obstacle remains: ABCB1 at the blood-brain barrier restricts HIV protease inhibitor uptake into the brain, thereby creating a sanctuary where the virus can persist and replicate (Kim et al., 1998a,b; Lee et al., 1998; Edwards et al., 2002). Virus replication in the brain causes decline in motor and cognitive function and can lead to dementia (Edwards et al., 2002). Further, antiretroviral drugs are a double-edged sword: They are effective against HIV in the periphery but increase *ABCB1* expression at the blood-brain barrier through activation of nuclear receptors such as PXR or CAR, thereby hindering drugs from reaching the brain (Chan et al., 2013). In a recent study, McRae et al. provided evidence that the HIV protein Tat1 increased *Abcb1* expression and Abcb1 activity levels at the blood-brain barrier in mouse models, further restricting brain uptake of antiretroviral drugs (McRae et al., 2019).

### F. Stroke

Stroke is one of the deadliest neurologic diseases causing annually more than 3 million deaths worldwide (Katan and Luft, 2018). Only 20% of patients who survive a stroke regain complete independence, making stroke the CNS disease responsible for the highest rate of long-term disability (WHO, 2006). In patients who survive, stroke is known to induce neuroinflammation and blood-brain barrier damage, mainly by disrupting tight junctions (Yang et al., 2019). Further, Dazert et al. showed in a rat ischemic stroke model that middle cerebral artery occlusion increases the expression of several ABC transporters, including Abcb1 and Abcg2, in the infarct region, potentially limiting brain uptake of neuroprotective drugs (Dazert et al., 2006). A follow-up study by DeMars et al. (2017) confirmed that Abcb1 protein levels are significantly increased after ischemic stroke in the brain capillary endothelium in vivo (DeMars et al., 2017). The presence of blood-brain barrier ABC transporters decreased drug concentrations in the ischemic brain by up to an order of magnitude, thus reducing neuroprotective drug efficacy (Spudich et al., 2006; Kilic et al., 2008; ElAli and Hermann, 2010). Inhibiting Abcb1 or silencing *Abcb1* with siRNA reduced levels of inflammatory cytokines, matrix metalloproteinases (membrane metalloprotease-2 and -9), and adhesion molecules (ICAM-1 and VCAM-1), which resulted in reduced infarct volume (Huang et al., 2022). These findings suggest that Abcb1 impairs brain drug entry of therapeutic drugs and may also contribute to barrier dysfunction in ischemic stroke and could potentially be a therapeutic target. More research is needed in this area to clarify the role of ABC transporters during stroke.

### G. Amyotrophic Lateral Sclerosis

ALS is a neurodegenerative disease leading to neuronal damage and loss of voluntary muscle movement. To this day, there is no cure, and only limited treatment options are available. Riluzole is one of two FDA-approved drugs for ALS therapy and is also an ABCB1 substrate, restricting riluzole brain uptake and efficacy (Jablonski et al., 2014). A preclinical study in a mouse ALS model showed that the Abcb1 inhibitor elacridar increases riluzole brain uptake and improves drug efficacy (Jablonski et al., 2014). Drug brain uptake is further restricted due to Abcb1 upregulation in brain capillary endothelial cells and in astrocytes of mice with a SOD1 mutation, a commonly used animal ALS model (Jablonski et al., 2012; Qosa et al., 2016b; Chan et al., 2017). ABCB1 expression increases in human pluripotent stem cell-derived brain endothelial cells that were cocultured with astrocytes isolated from ALS patient samples (Mohamed et al., 2019).

## V. Concluding Remark

CNS disorders make up approximately 50% of the total health burden in the United States, significantly contributing to mortality and disability (Gooch et al., 2017). Moreover, CNS disorders are often difficult to treat (Gooch et al., 2017), which is in part because many CNS active drugs are substrates of ABCB1 and ABCG2 at the blood-brain barrier. We now know that ABCB1 and ABCG2, and possibly other ABC transporters, work together in restricting brain drug uptake, rendering CNS pharmacotherapy extremely difficult. Over the past decades, numerous therapeutic approaches have been tested to overcome blood-brain barrier efflux transporters and to improve treatment outcomes in patients with CNS disorders. In this regard, we have worked to unravel signaling pathways that regulate transporters (Hartz and Bauer, 2010). Part of this research involves identifying target molecules that can be manipulated to control ABC transporter expression and/or activity. For example, this approach could be used to increase transporter expression and/or activity to protect the brain while treating peripheral diseases when CNS effects are not desired (Bauer et al., 2004, 2007). Consider the chemotherapy of peripheral cancers, where one prominent side effect is the development of “chemo brain,” a form of drug-induced dementia (Joshi et al., 2010; Ren et al., 2019; Stefancin et al., 2020). In this case, shielding the brain from anticancer drugs by upregulating ABC transporters could be beneficial to prevent chemo brain, which would improve patients’ overall health and quality of life.

On the other hand, targeting transporter regulation has the potential to open the barrier for a short “window in time” and allow drug uptake when needed while protecting the brain in between treatments (Hartz and Bauer, 2010). Selectively turning off ABC transporters to increase brain uptake of CNS therapeutics could be an important tool to improve the treatment of various CNS disorders (Hartz and Bauer, 2010). Since reducing ABC transporter activity could exacerbate conditions such as Alzheimer’s disease, including prodromal stages, where ABCB1 activity is critical for clearance of A*β* from the brain, chronic downregulation of ABC transporters could bear risks. This further supports the idea of time-limited ABCB1/ABCG2 reduction to maximize drug delivery and therapeutic outcomes, while minimizing risk that could stem from chronic transporter suppression. Several of the pathways described here could be targeted with existing FDA-approved drugs with the potential of regulating *ABCB1*/*ABCG2* expression and associated proteins’ activity at the blood-brain barrier (Table 1). For example, ER-mediated decrease of *ABCB1*/*ABCG2* expression and associated proteins activity levels at the blood-brain barrier could potentially be accomplished with ethinyl estradiol or could be blocked with fulvestrant (Imai et al., 2002, 2005; Hartz et al., 2010a,b; Zuloaga et al., 2012). Inflammation-mediated changes in ABCB1/ABCG2 could be blocked with the anti-TNF*α* antibodies adalimumab or infliximab or with the cyclooxygenase 2 inhibitor celecoxib to prevent *ABCB1* and *ABCG2* upregulation (Hartz et al., 2004, 2006; Bauer et al., 2007; Zibell et al., 2009). Another option is the use of tyrosine kinase inhibitors such as lapatinib (EGFR), erlotinib (EGFR), sunitinib (VEGFR), or bevacizumab (VEGFR), to decrease ABCB1/ABCG2 expression/activity levels at the blood-brain barrier (Evseenko et al., 2007; Hawkins et al., 2010; Tome et al., 2015). While this approach seems promising, more research is needed to evaluate the effect of increased or decreased ABC transporter expression and activity at the blood-brain barrier on drug brain levels, efficacy, and overall disease progression.

**TABLE 1:** Examples of FDA-approved drugs that could downregulate ABCB1/ABCG2 and provide a window-in-time for brain drug delivery

Target	Drug Name	Drug Action	Predicted Outcome
COX-2	Fenoprofen	Inhibitor	ABCB1 ↓
COX-2	Acetic salicylic acid	Inhibitor	ABCB1 ↓
COX-2	Diclofenac	Inhibitor	ABCB1 ↓
COX-2	Celecoxib	Inhibitor	ABCB1 ↓
COX-2	Bromfenac	Inhibitor	ABCB1 ↓
COX-2	Etoricoxib	Inhibitor	ABCB1 ↓
COX-2	Firocoxib	Inhibitor	ABCB1 ↓
COX-2	Flurbiprofen	Inhibitor	ABCB1 ↓
COX-2	Ibuprofen	Inhibitor	ABCB1 ↓
COX-2	Indomethacin	Inhibitor	ABCB1 ↓
COX-2	Ketorolac	Inhibitor	ABCB1 ↓
COX-2	Meloxicam	Inhibitor	ABCB1 ↓
COX-2	Nabumetone	Inhibitor	ABCB1 ↓
COX-2	Naproxen	Inhibitor	ABCB1 ↓
COX-2	Oxaprozin	Inhibitor	ABCB1 ↓
COX-2	Parecoxib	Inhibitor	ABCB1 ↓
COX-2	Piroxicam	Inhibitor	ABCB1 ↓
COX-2	Tenoxicam	Inhibitor	ABCB1 ↓
EGFR	Cetuximab	EGFR neutralizing antibody	ABCB1 ↓; ABCG2 ↓
EGFR	Erlotinib	Inhibitor	ABCB1 ↓; ABCG2 ↓
EGFR	Lapatinib	Inhibitor	ABCB1 ↓; ABCG2 ↓
EGFR	Mobocertinib	Inhibitor	ABCB1 ↓; ABCG2 ↓
EGFR	Necitumumab	EGFR neutralizing antibody	ABCB1 ↓; ABCG2 ↓
EGFR	Osimertinib	Inhibitor	ABCB1 ↓; ABCG2 ↓
EGFR	Panitumumab	EGFR neutralizing antibody	ABCB1 ↓; ABCG2 ↓
EGFR	Gefitinib	Inhibitor	ABCB1 ↓; ABCG2 ↓
ER	Estradiol	Agonist	ABCB1 ↓; ABCG2 ↓
ER	Estramustine	Agonist	ABCB1 ↓; ABCG2 ↓
ER	Ethinyl estradiol	Agonist	ABCB1 ↓; ABCG2 ↓
ER	Norethisterone	Agonist	ABCB1 ↓; ABCG2 ↓
ER	Norethynodrel	Agonist	ABCB1 ↓; ABCG2 ↓
GR	Mifepristone	Antagonist	ABCB1 ↓; ABCG2 ↓
GR	Ulipristal	Antagonist	ABCB1 ↓; ABCG2 ↓
MR	Fineronone	Antagonist	ABCB1 ↓; ABCG2 ↓
NMDAR	Esketamine	Antagonist	ABCB1 ↓
NMDAR	Memantine	Antagonist	ABCB1 ↓
mTOR	Everolimus	Inhibitor	ABCB1 ↓; ABCG2 ↓
mTOR	Sirolimus	Inhibitor	ABCB1 ↓; ABCG2 ↓
mTOR	Temsirolimus	Inhibitor	ABCB1 ↓; ABCG2 ↓
PI3K	Alpelisib	Inhibitor	ABCB1 ↓; ABCG2 ↓
PI3K	Copanlisib	Inhibitor	ABCB1 ↓; ABCG2 ↓
PI3K	Duvelisib	Inhibitor	ABCB1 ↓; ABCG2 ↓
TNF*α*	Adalimumab	TNF*α* neutralizing antibody	ABCB1 ↓
TNF*α*	Certolizumab	TNF*α* neutralizing antibody	ABCB1 ↓
TNF*α*	Etanercept	TNF*α* inhibitor	ABCB1 ↓
TNF*α*	Golimumab	TNF*α* neutralizing antibody	ABCB1 ↓
TNF*α*	Infliximab	TNF*α* neutralizing antibody	ABCB1 ↓

Materials and FDA-approval status were determined based on the FDALabel database in September 2021 (https://www.fda.gov/science-research/bioinformatics-tools/fdalabel-full-text-search-drug-product-labeling), specifically Section 12.1, “Mechanism of Action.”

## Abbreviations

3′-UTR: 3′-untranslated region
A*β*: amyloid *β*
ABC: ATP-binding cassette
AhR: aryl hydrocarbon receptor
ALS: amyotrophic lateral sclerosis
CAR: constitutive androstane receptor
CNS: central nervous system
CSF: cerebral spinal fluid
E2: 17*β*-estradiol
DIPG: diffuse intrinsic pontine glioma
EGF: epidermal growth factor
EGFR: epidermal growth factor receptor
ER: estrogen receptor
ERK: extracellular signal-regulated kinase
FDA: Food and Drug Administration
FXR: Farnesoid X receptor
GR: glucocorticoid receptor
HDAC: histone acetyltransferase and histone deacetylase
HIV: human immunodeficiency virus
IL: interleukin
JAK-STAT3: Janus kinase and signal transducer and activator of transcription 3
LXR: liver X receptor
MAPK: p38 mitogen-activated protein kinase
miRNA: micro RNA
mRNA: messenger RNA
NBD: nucleotide binding domains
NF-*κ*B: nuclear factor kappa-light-chain-enhancer of activated B cells
NMDAR: N-methyl-D-aspartate receptor
NO: nitric oxide
Nrf2: nuclear factor E2-related factor 2
PPAR: peroxisome proliferator-activated receptor
PXR: pregnane X receptor
RAR: retinoid acid receptor
ROS: reactive oxygen species
RTK: receptor tyrosine kinase
RXR: retinoid X receptor
siRNA: small interfering RNA
SNP: single nucleotide polymorphisms
T4: thyroxin
TMD: transmembrane domain
TNF*α*: tumor necrosis factor *α*
VDR: vitamin D receptor
VEGF: vascular endothelial growth factor
WHO: World Health Organization
